# Supplementary material for: Convergence of distinct signaling pathways on synaptic scaling to trigger rapid antidepressant action
Source: Cell Rep. Author manuscript; Available in PMC 2021 Nov 13. (PMC8590465; doi:10.1016/j.celrep.2021.109918)
Supplement: 1 [file NIHMS1753766-supplement-1.pdf]

Cell Reports, Volume *37*

Supplemental information

Convergence of distinct signaling pathways on synaptic scaling to trigger rapid antidepressant action

Kanzo Suzuki, Ji-Woon Kim, Elena Nosyreva, Ege T. Kavalali, and Lisa M. Monteggia

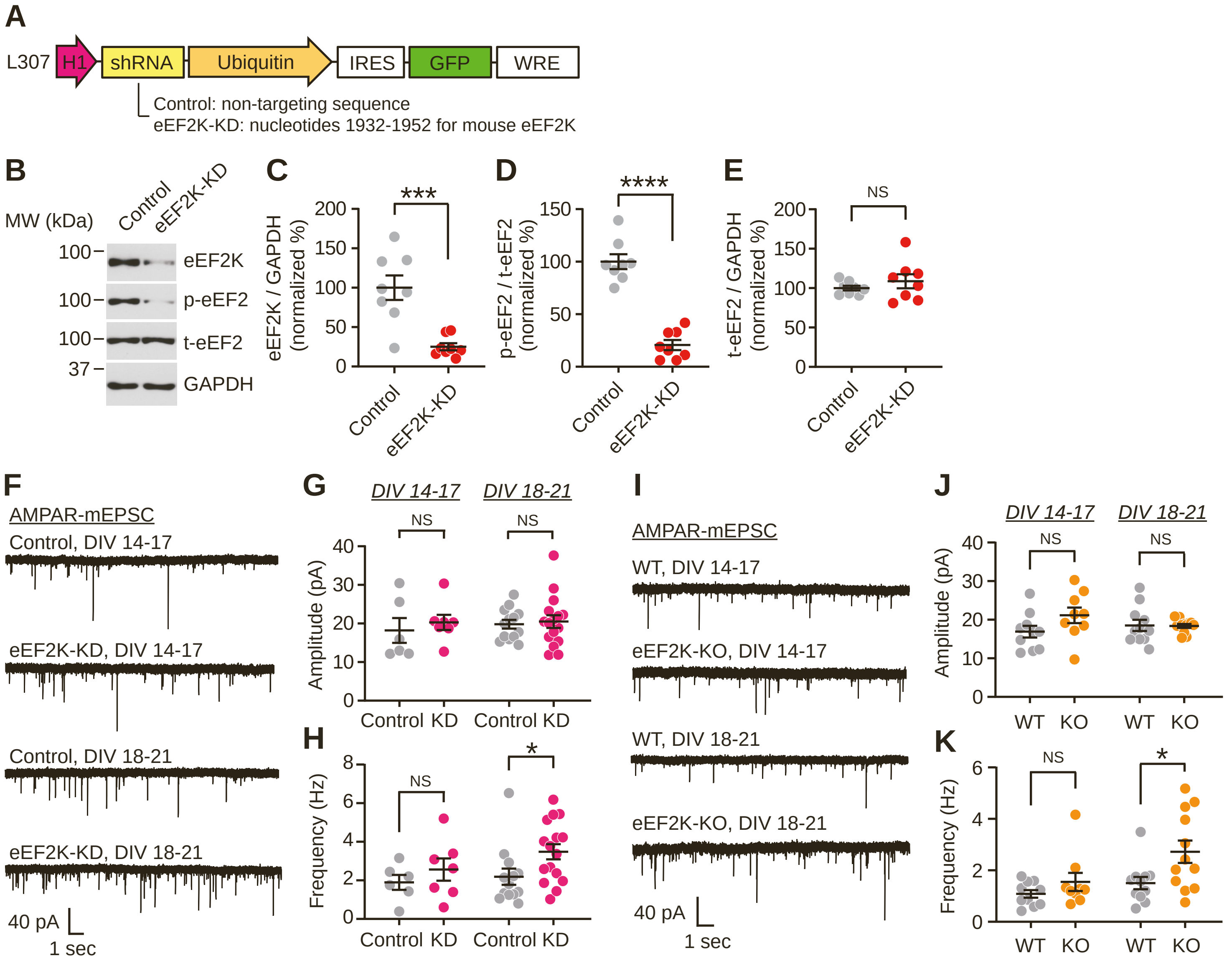

Fig. S1. Characterization of eEF2K knockdown and the effect on AMPAR-mEPSCs in cultured hippocampal neurons, related to Figure 1.
(A) Diagram of knockdown construct for eEF2K. shRNA sequence for corresponding to nucleotides 1932-1952 against mice eEF2K was subcloned into L307 vector. Non-targeting shRNA sequence was used as a control construct. (B) To confirm knockdown efficiency of eEF2K knockdown construct, control or eEF2K-KD lentivirus were infected in cultured hippocampal neurons at DIV4. Cell lysate was made at DIV16-17 and western blotting (WB) was performed using antibodies against eEF2K, phosphorylated eEF2 (p-eEF2) and total eEF2 (t-eEF2). (C) eEF2K expression was significantly decreased to 25.1 % ± 4.5% by eEF2K-KD. (Unpaired t test, *P*=0.0004, *n*=8 in control and eEF2K-KD). (D) Phospho-eEF2 level was strongly decreased to 20.7% ± 4.8% by eEF2K-KD since eEF2 is unique substrate for eEF2K (Unpaired t test, *P*<0.0001, *n*=8 in control and eEF2K-KD). (E), Total eEF2 level was not changed by eEF2K-KD (Unpaired t test, *P*=0.367, *n*=8 in control and eEF2K-KD). (F) Representative traces of AMPA-mEPSC from cultured hippocampal neurons infected with lentivirus control or eEF2K- KD. The ages of culture are indicated. (G) AMPAR-mEPSC amplitude was similar between control and eEF2K-KD neurons in younger (DIV 14-17) and older (DIV 18-21) mature cultured hippocampal neurons (Unpaired t test, *P*=0.5798, *n*=6 in control neurons and *n*=7 in eEF2K-KD neurons at DIV 14-17; *P*=0.7437, *n*=13 in control neurons and 16 in eEF2K-KD neurons at DIV 18-21). (H) A significant increase of frequency was not observed in eEF2K-KD young culture but older eEF2K-KD neurons augmented frequency (Unpaired t test, *P*=0.3763, *n*=6 in control neurons and *n*=7 in eEF2K-KD neurons at DIV 14-17; *P*=0.0329, *n*=13 in control neurons and *n*=16 in eEF2K-KD neurons at DIV 18-21). (I) Representative traces of AMPAR-mEPSC from WT and eEF2K-KO cultured hippocampal neurons. The ages of culture are indicated. (J) AMPAR-mEPSC amplitude was similar between WT and eEF2K-KO neurons at DIV 14-17 and DIV 18-21 (Unpaired t test, *P*=0.1085, *n*=10 in WT and *n*=9 in eEF2K-KO neurons at DIV 14-17; *P*=0.9297, *n*=11 in WT neurons and *n*=12 in eEF2K-KO neurons at DIV 18-21). (K) Older mature eEF2K-KO neurons, not young neurons, significantly increased AMPAR-mEPSC frequency (Unpaired t test, *P*=0.223, *n*=10 in WT and *n*=9 in eEF2K-KO neurons at DIV 14-17; *P*=0.0258, *n*=11 in WT neurons and *n*=12 in eEF2K-KO neurons at DIV 18-21). Data are represented as mean ±SEM.

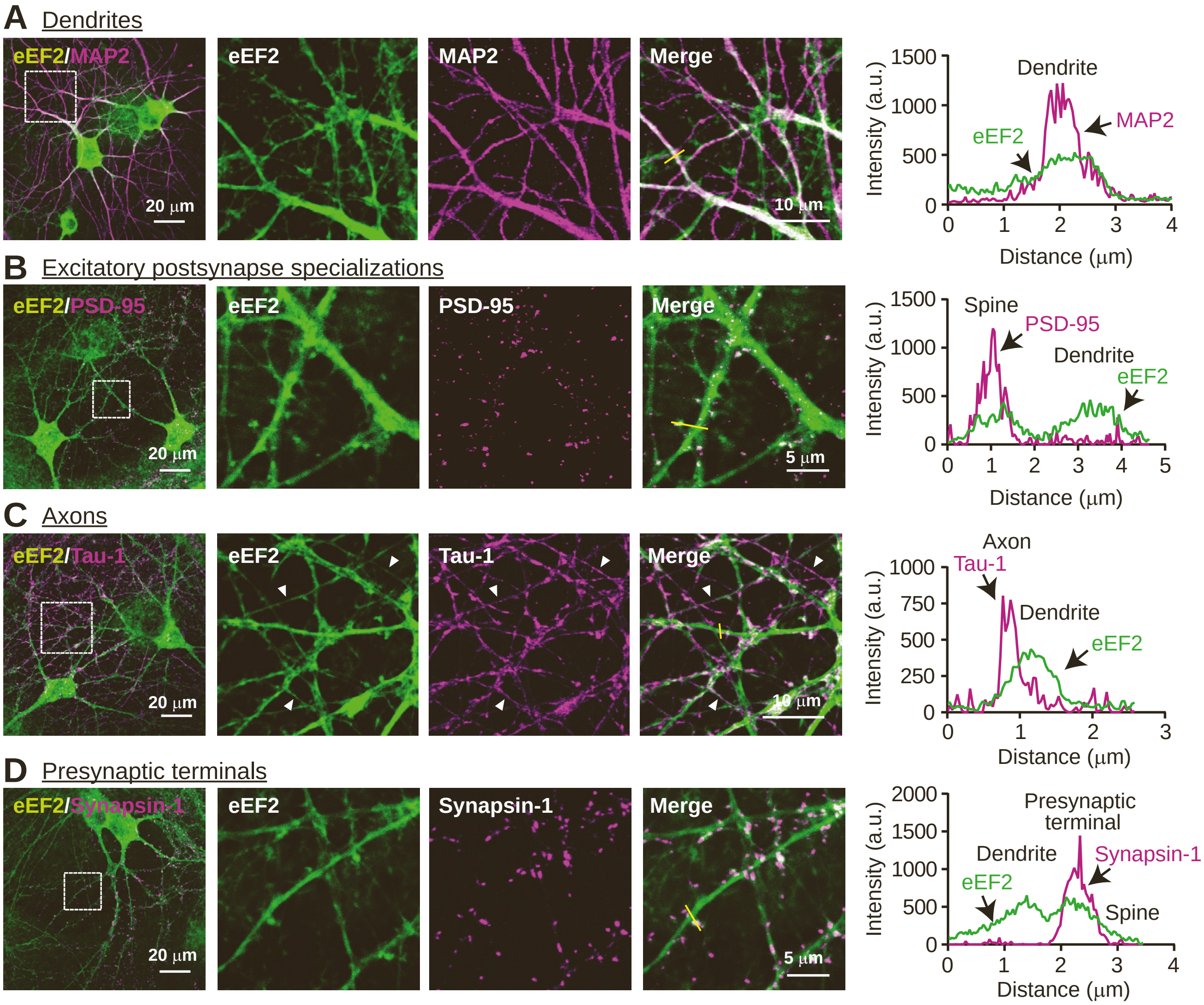

Fig. S2. Localization of eEF2 in cultured hippocampal neurons, related to Figure 1.
(A-D) To confirm the eEF2 distribution, immunostaining was performed using anti-total-eEF2. Left, Representative images of eEF2 with MAP2 (A), PSD-95 (B), tau-1 (C) or synapsin-1 (D) in mature cultured hippocampal neurons. Right, relative immunoreactivities (IR) on the yellow line in the merge figure (A-D). eEF2 was detected in dendrites and excitatory postsynapse specializations (A-B). eEF2 was observed in some axons (arrow head) but high eEF2 IR was detected in dendrites (C). Localization of eEF2 was not apparent in synapsin-I positive presynaptic terminal on dendrites (D). Scale bar, 20 μm, 10 μm or 5 μm.

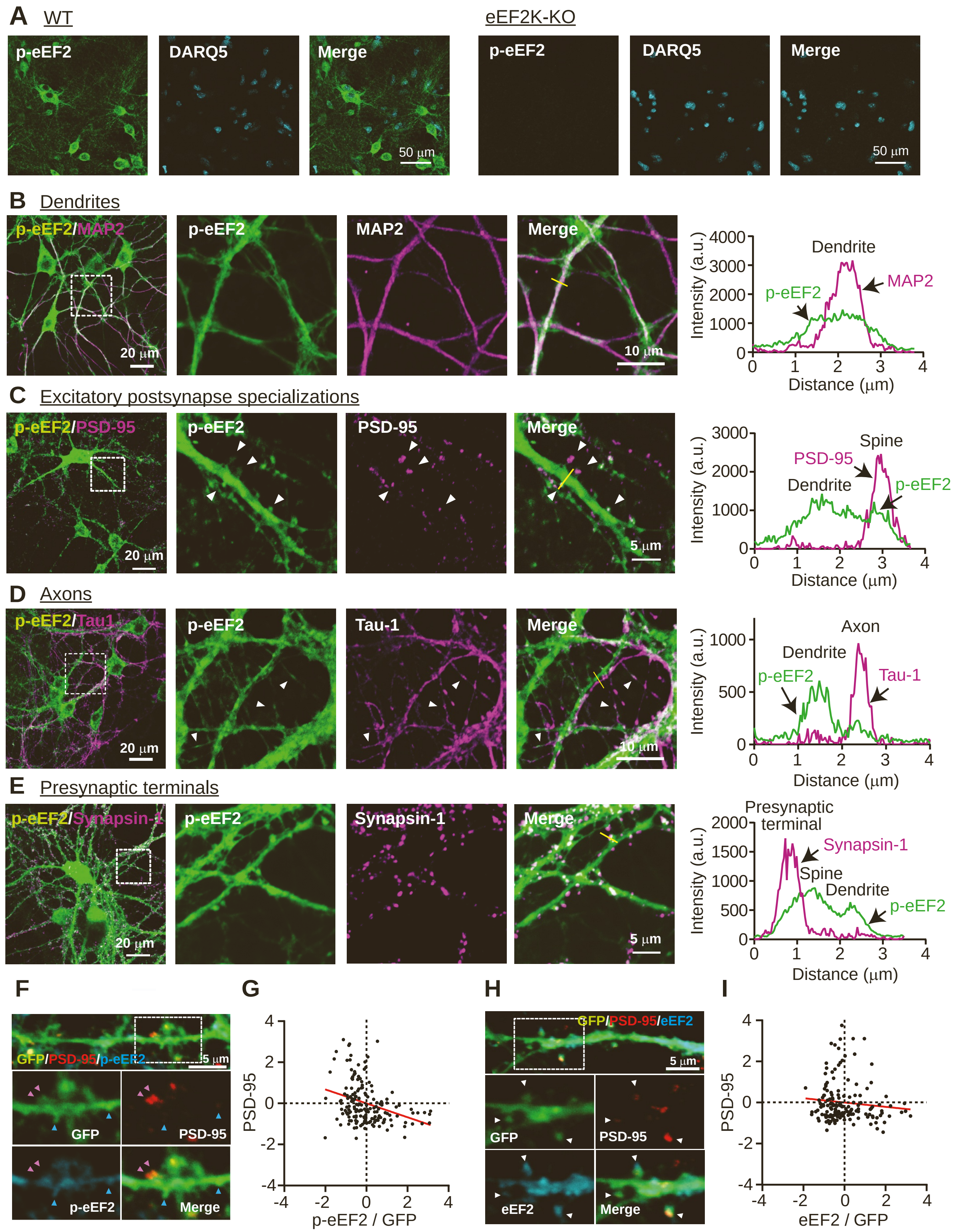

A
WT
p-eEF2
DARQ5
Merge
50 μm
eEF2K-KO
p-eEF2
DARQ5
Merge
50 μm
B
Dendrites
p-eEF2/MAP2
20 μm
p-eEF2
MAP2
Merge
10 μm
Intensity (a.u.)
4000
3000
2000
1000
0
Dendrite
MAP2
p-eEF2
Distance (μm)
C
Excitatory postsynapse specializations
p-eEF2/PSD-95
20 μm
p-eEF2
PSD-95
Merge
5 μm
Spine
PSD-95
Dendrite
p-eEF2
Intensity (a.u.)
Distance (μm)
D
Axons
p-eEF2/Tau1
20 μm
p-eEF2
Tau-1
Merge
10 μm
Axon
Dendrite
p-eEF2
Tau-1
Intensity (a.u.)
Distance (μm)
E
Presynaptic terminals
p-eEF2/Synapsin-1
20 μm
p-eEF2
Synapsin-1
Merge
5 μm
Presynaptic terminal
Synapsin-1
Spine
Dendrite
p-eEF2
Intensity (a.u.)
Distance (μm)
F
GFP/PSD-95/p-eEF2
5 μm
GFP
PSD-95
p-eEF2
Merge
G
PSD-95
p-eEF2 / GFP
H
GFP/PSD-95/eEF2
5 μm
GFP
PSD-95
eEF2
Merge
I
PSD-95
eEF2 / GFP

Fig. S3. eEF2 is phosphorylated in dendrites and excitatory postsynaptic spines, related to Figure 1.

(A) To confirm anti-phospho-eEF2 antibody specificity, immunostaining of cultured WT or eEF2K-KO hippocampal neurons was performed using antibody against phosphorylated eEF2 (p-eEF2). Immunoreactivity against p-eEF2 was abolished in eEF2K-KO hippocampal neurons. Nuclei were detected by DARQ5. Scale bar, 50 μm. (B-E) Left, Representative images of phosphorylated eEF2 (p-eEF2) with MAP2 (B), PSD-95 (C), tau-1 (D) or synapsin-1 (E) in mature cultured hippocampal neurons. Right, relative immunoreactivities (IR) on the yellow line in the merge figure (B-E). According to MAP2 or PSD-95 staining, eEF2 phosphorylation was observed in dendrites and excitatory postsynapse specializations (B-C). Unexpectedly, we observed variation of p-eEF2 IR and PSD-95 IR in synapses (arrow head in C). PSD-95 enrichment was detected in the spine showing lower eEF2 phosphorylation (C). Axons were identified using tau-1 staining in cultured hippocampal neurons. p-eEF2 IR was observed in some parts of axon (arrow head) (D). eEF2 phosphorylation was not apparent in synapsin-I positive presynaptic terminal on dendrites (E). Scale bar, 20 μm, 10 μm or 5 μm. (F) To closely analyze eEF2 phosphorylation level in the dendritic spines, we performed immunocytochemistry for phosphorylated (p-eEF2) with PSD-95 in the GFP-expressing hippocampal neurons. Several spines showed strong PSD-95 IR but they showed small p-eEF2 IR (pink arrow head). Conversely, small PSD-95 IR was observed in the spines showing high eEF2 phosphorylation (blue arrow head). Scale bar, 5 μm. (G) Relative eEF2 phosphorylation (p-eEF2/GFP) in the spines was determined by normalization of p-eEF2 IR with GFP IR as GFP is a volume marker. Then, individual p-eEF2/GFP and PSD-95 values were plotted in scatter plot as average is 0 and standard deviation is 1. Correlation analysis revealed small negative correlation between PSD-95 and relative p-eEF2 in the spines (Pearson correlation, r=-0.3378, P<0.0001 from 7 dendrites). (H) we performed same analysis using eEF2 staining to make sure whether this inverse correlation was occurred by eEF2 phosphorylation, not eEF2 expression. eEF2 was detected in PSD-95 positive spines (white arrow head). Scale bar, 5 μm. (I) PSD-95 intensities were not correlated with relative eEF2 intensities (Pearson correlation, r=-0.1049, P=0.1941 from 7 dendrites).

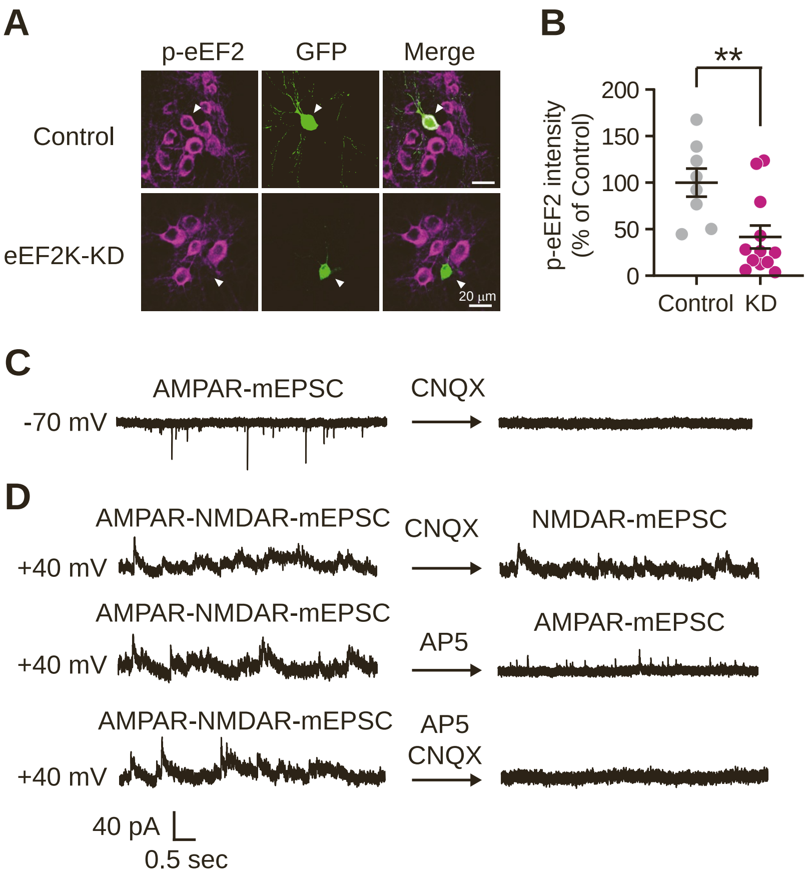

Fig. S4. Single cell knockdown for eEF2K and pharmacological isolation of AMPAR-mEPSCs and NMDAR-mEPSCs, related to Figure 1.

(A) Representative images of phosphorylated eEF2 (p-eEF2) in the cultured hippocampal neurons transfected with control or eEF2K-KD construct. Neurons were fixed at 3-4 day after transfection and immunocytochemistry for p-eEF2 was performed. p-eEF2 IR was reduced in the single cell eEF2K knockdown hippocampal neuron (arrow head). Scale bar, 20 µm. (B) p-eEF2 intensities in soma were measured in control or eEF2K-KD neurons. p-eEF2 signal was significantly reduced in the eEF2K-KD neurons (Unpaired t test, P=0.0077, n=8 in control and n=12 in eEF2K-KD). (C) AMPAR-mEPSC was isolated in the presence of Mg^{2+} at -70 mV since Mg^{2+} blocks NMDAR at -70 mV. Indeed, CNQX eliminated peaks of AMPAR-mEPSC. (D) AMPAR-NMDAR-mEPSC was recoding at +40 mV since Mg^{2+} is released from NMDAR at +40 mV. NMDA-mEPSC was isolated in the presence of CNQX. AMPAR-mEPSC was detected in the presence of AP-5 at +40 mV. Peaks were not detected in the presence of AP-5 and CNQX. Data are represented as mean ±SEM.

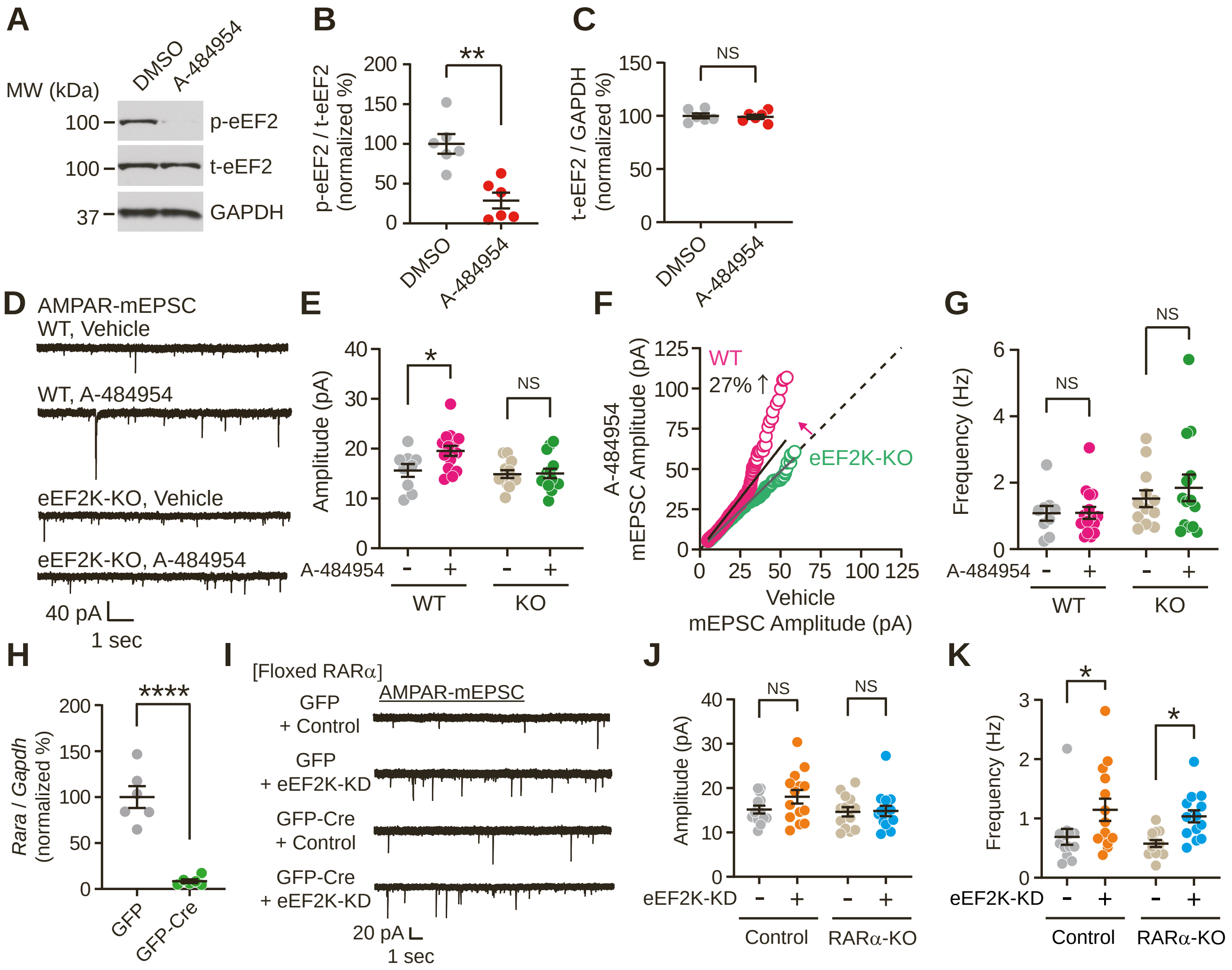

Fig. S5. Characterization of A484954-mediated synaptic scaling in eEF2K knockout neurons and the effect of loss of eEF2K on AMPAR-mEPSCs in RARα knockout neurons, related to Figure 2 and 3.

(A) To confirm whether A-484954 inhibits eEF2K activity in hippocampal neurons, we performed WB against phosphorylated eEF2 (p-eEF2) or total eEF2 (t-eEF2). After incubation of 30 μM A-484954 for 1 hour in wild type hippocampal culture, neuronal cells were lysed in lysis buffer and WB was performed using p-eEF2 and t-eEF2 antibodies. (B) Phosphorylated eEF2 was significantly reduced to 28.7 % ± 9.9% by A-484954 (Unpaired t test, *P*=0.0011, *n*=6 in control and eEF2K-KD). (C) Total EF2 level was not changed by A-484954 treatment (Unpaired t test, *P*=0.7719, *n*=6 in control and eEF2K-KD). (D) Representative traces of AMPAR-mEPSC from WT or eEF2K-KO cultured hippocampal neurons treated with vehicle or 30 μM A-484954 for 1 hour. (E) Acute treatment of A-484954 significantly increased AMPAR-mEPSC amplitude in WT neurons but eEF2K-KO neurons occluded it, suggesting that A-484954-mediated synaptic scaling acts through eEF2K dependent (Two-way ANOVA with Sidak's multiple comparisons, Vehicle vs A-484954 in WT *P*=0.0229, Vehicle vs A-484954 in eEF2K-KO *P*=0.9938, *n*=9-15 per group). (F) Rank order plot for AMPAR-mEPSC amplitude with or without A-484954 treatment in WT and eEF2K-KO neurons (Linear regression, slope= 1.27 in WT and =0.97 in eEF2K-KO). (G) Treatment of A-484954 did not change AMPAR-mEPSC frequency (Two-way ANOVA with Sidak's multiple comparisons, Vehicle vs A-484954 in WT *P*=0.9996, Vehicle vs A-484954 in eEF2K-KO *P*=0.6665, *n*=9-15 per group). (H) To confirm the RARα expression level in cultured floxed RARα hippocampal neurons with Cre lentivirus infection, mRNA was collected and RT-PCR was performed. RARα mRNA expression was significantly decreased to 8.445 ± 1.951 % by Cre lentivirus infection (Unpaired t test, *P*<0.0001, *n*=6 in GFP and GFP-Cre). (I) Effect of lentiviral eEF2K-KD on AMPAR-mEPSCs was confirmed in RARα-KO culture at DIV 18-21. (J) eEF2K-KD did not alter AMPAR-mEPSC amplitude in control and RARα-KO neurons (Two-way ANOVA with Sidak's multiple comparisons, control vs eEF2K-KD in control *P*=0.1824, control vs eEF2K-KD in RARα-KO *P*=0.9908, *n*=13-14 per group). (K) eEF2K-KD significantly increased AMPAR-mEPSC frequency even in RARα-KO neurons (Two-way ANOVA with Sidak's multiple comparisons, control vs eEF2K-KD in control *P*=0.0349, Control vs eEF2K-KD in RARα-KO *P*=0.0340, *n*=13-14 per group). Data are represented as mean ±SEM.

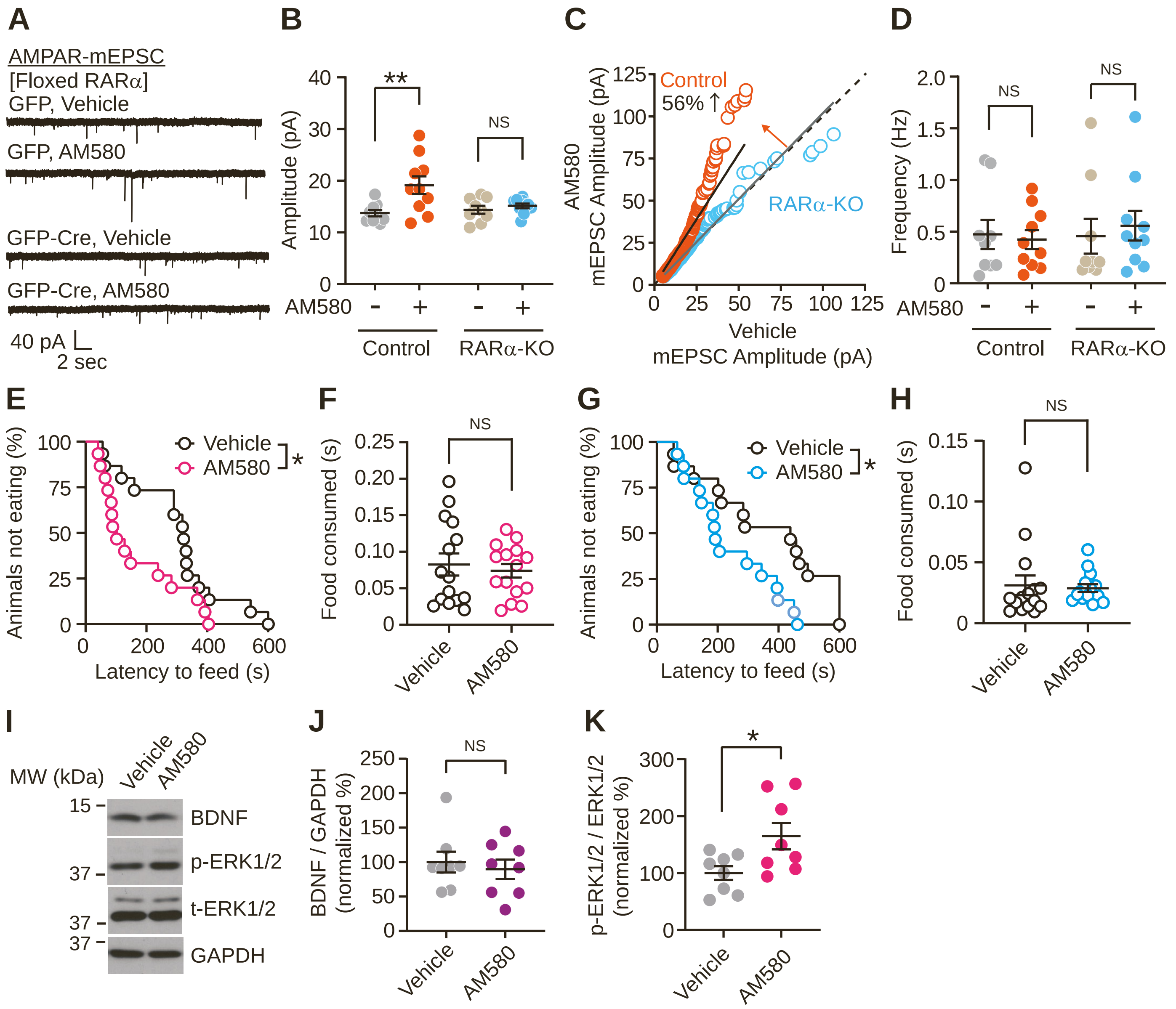

Fig. S6. The effect of RARα agonist AM580 on synaptic scaling, novelty-suppressed feeding and downstream signaling, related to Figure 4.

(A) Representative traces of AMPAR-mEPSC from control or RARα-KO cultured hippocampal neurons treated with vehicle or 10 μM AM580 for 1 hour. (B) Acute treatment of AM580 significantly increased AMPAR-mEPSC amplitude in control neurons but RARα-KO neurons occluded it, indicating AM580 specifically acts on RARα (Two-way ANOVA with Sidak's multiple comparisons, Vehicle vs AM580 in control P=0.0018, Vehicle vs AM580 in RARα-KO P=0.8507, n=9-10 per group). (C) Rank order plot for AMPAR-mEPSC amplitude with or without AM580 treatment in control and RARα-KO neurons (Linear regression, slope=1.56 in control and =1.02 in RARα-KO). (D) Treatment of A-484954 did not change AMPAR-mEPSC frequency (Two-way ANOVA with Sidak's multiple comparisons, Vehicle vs AM580 in control P=0.9592, Vehicle vs AM580 in RARα-KO P=0.8428, n=9-10 per group). (E) C57BL/6 mice were administered vehicle or AM580 (20 mg/kg) and assessed 2 hours later in novelty-suppressed feeding (NSF). AM580-treated C57BL/6 mice showed a significant decrease in latency to feed compared with vehicle (Log-rank test, P=0.0373, n=15 in vehicle and AM580). (F) appetite post-test following the NSF showed that the total amount of food consumed was similar between vehicle- and AM580-treated mice, ruling out a possible confound to the NSF test (Unpaired t test, P=0.6304, n=15 in vehicle and AM580). (G-H) 7 days after AM580 administration, AM580-treated mice displayed antidepressant-like response in the NSF (Log-rank test, P=0.0140, n=15 in vehicle and AM580), with no significant effect on food consumption test (Unpaired t test, P=0.7956, n=15 in vehicle and AM580). (I) To confirm whether AM580 increases BDNF or ERK phosphorylation in hippocampus, we quantified BDNF and ERK phosphorylation 2 hours after administration of vehicle or AM580 (20 mg/kg) in mice. Hippocampus was dissected and lysed in lysis buffer. WB was performed using antibodies [anti-BDNF, anti-phospho-EFK1/2 (p-ERK1/2), anti-total-ERK1/2 (t-ERK1/2) or anti-GAPDH]. (J) BDNF level was not altered by AM580 treatment (Unpaired t test, P=0.6242, n=8 in vehicle and AM580). (K) phosphorylation of ERK was significantly increased by AM580 treatment (Unpaired t test, P=0.0269, n=8 in vehicle and AM580). Data are represented as mean ±SEM.

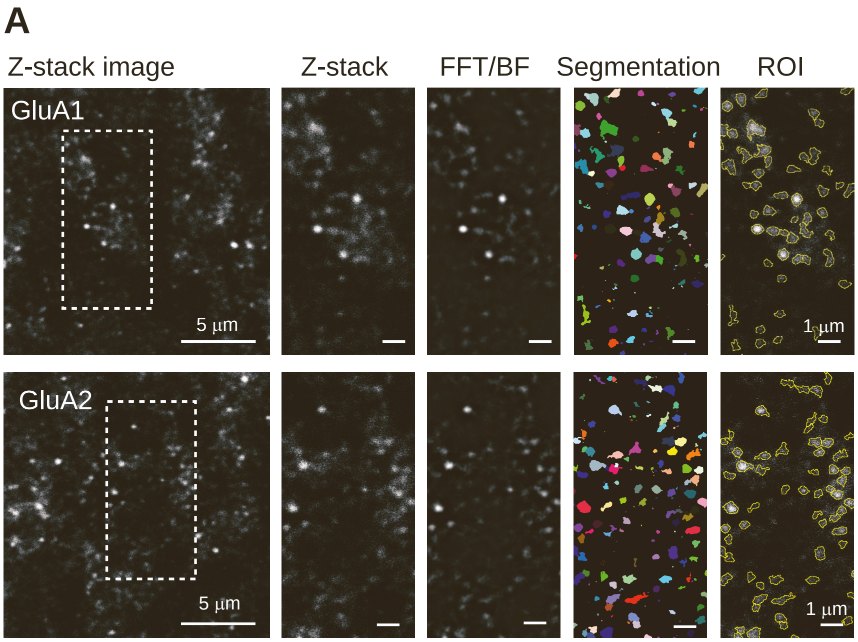

Fig. S7. Illustration of optical analysis for surface GluA1 and GluA2 puncta, related to Figure 5.
(A) To enhance particle contrast, surface GluA1 and GluA2 images of stratum radiatum of hippocampus CA1 region (as shown in Figure 5A and 5B) were processed using fast Fourier transform (FFT) bandpass filter (BF). To segment adjacent puncta, interactive H-watershed was applied on FFT/BF image. Regions of interest (ROIs) were determined using particle analysis. Area criteria was set to 0.05-1.00 μm^2. Area size and intensity in individual puncta were obtained from Z-stack image. Scale bar, 5 µm or 1 µm.
