# Supplementary material for: Convergence of distinct signaling pathways on synaptic scaling to trigger rapid antidepressant action
Source: Cell Rep. Author manuscript; Available in PMC 2021 Nov 13. (PMC8590465; doi:10.1016/j.celrep.2021.109918)
Supplement: 2 [file NIHMS1753766-supplement-2.pdf]

Cell Reports

Article

Convergence of distinct signaling pathways on synaptic scaling to trigger rapid antidepressant action

Graphical abstract

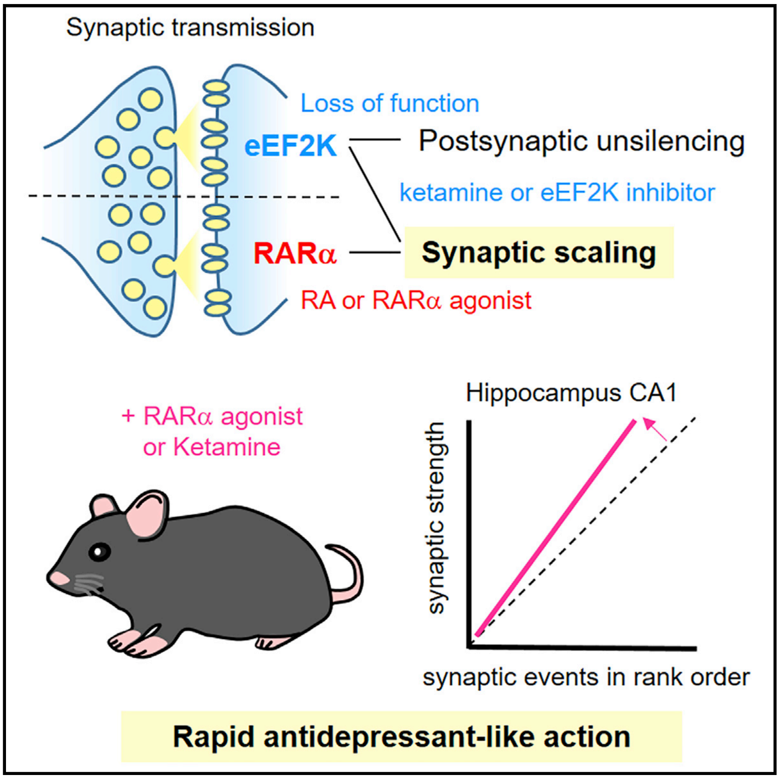

Highlights

- Acute suppression of eEF2K elicits synaptic scaling
- A similar form of synaptic scaling is rapidly elicited by retinoic acid
- These two independent pathways converge at a common synaptic endpoint
- Multiplicative synaptic scaling is sufficient to produce rapid antidepressant effects

Authors

Kanzo Suzuki, Ji-Woon Kim, Elena Nosyreva, Ege T. Kavalali, Lisa M. Monteggia

Correspondence

ege.kavalali@vanderbilt.edu (E.T.K.), lisa.monteggia@vanderbilt.edu (L.M.M.)

In brief

Suzuki et al. examine the role of eEF2K and retinoic acid signaling pathways in synaptic plasticity and rapid antidepressant effects. Their study establishes a causal link between synaptic scaling and rapid antidepressant effects and proposes that this form of synaptic plasticity is a key synaptic substrate for rapid antidepressant action.

Suzuki et al., 2021, Cell Reports *37*, 109918
November 2, 2021 © 2021 The Author(s).
https://doi.org/10.1016/j.celrep.2021.109918

CellPress

Cell Reports

CellPress
OPEN ACCESS

Article

Convergence of distinct signaling pathways on synaptic scaling to trigger rapid antidepressant action

Kanzo Suzuki,[1] Ji-Woon Kim,[1] Elena Nosyreva,[2] Ege T. Kavalali,[1,*] and Lisa M. Monteggia[1,3,*]

[1]Department of Pharmacology and the Vanderbilt Brain Institute, Vanderbilt University, Nashville, TN 37232, USA
[2]Department of Neuroscience, University of Texas Southwestern Medical Center, Dallas, TX 75390-9111, USA
[3]Lead contact
*Correspondence: ege.kavalali@vanderbilt.edu (E.T.K.), lisa.monteggia@vanderbilt.edu (L.M.M.)
https://doi.org/10.1016/j.celrep.2021.109918

SUMMARY

Ketamine is a noncompetitive glutamatergic *N*-methyl-D-aspartate receptor (NMDAR) antagonist that exerts rapid antidepressant effects. Preclinical studies identify eukaryotic elongation factor 2 kinase (eEF2K) signaling as essential for the rapid antidepressant action of ketamine. Here, we combine genetic, electrophysiological, and pharmacological strategies to investigate the role of eEF2K in synaptic function and find that acute, but not chronic, inhibition of eEF2K activity induces rapid synaptic scaling in the hippocampus. Retinoic acid (RA) signaling also elicits a similar form of rapid synaptic scaling in the hippocampus, which we observe is independent of eEF2K functioni. The RA signaling pathway is not required for ketamine-mediated antidepressant action; however, direct activation of the retinoic acid receptor α (RARα) evokes rapid antidepressant action resembling ketamine. Our findings show that ketamine and RARα activation independently elicit a similar form of multiplicative synaptic scaling that is causal for rapid antidepressant action.

INTRODUCTION

Major depressive disorder is one of the most common and serious mental disorders. Traditional antidepressants such as tricyclic antidepressants (TCAs) and monoamine oxidase inhibitors (MAOIs), as well as later-generation drugs, including serotonin-selective reuptake inhibitors (SSRI) and serotonin-norepinephrine reuptake inhibitors (SNRIs), acutely increase monoaminergic neurotransmission and yet typically take several weeks to exert antidepressant responses. This therapeutic delay in onset is a limitation of traditional antidepressant therapies, especially for individuals at risk for suicide. Thus, there is tremendous need for pharmacological therapies that can quickly ameliorate symptoms associated with depression, including treatment-resistant depression.

Ketamine is a noncompetitive glutamatergic *N*-methyl-D-aspartate receptor (NMDAR) antagonist that exerts rapid and robust antidepressant efficacy in patients with major depression, including treatment-resistant depression (Berman et al., 2000; Daly et al., 2018; Price et al., 2009; Zarate et al., 2006). The antidepressant action of ketamine has been hypothesized to occur by the block of NMDARs, driven by spontaneous synaptic transmission, that results in the inhibition of the calcium/calmodulin-dependent protein kinase, eukaryotic elongation factor 2 kinase (eEF2K), which through its downstream effects desuppresses protein synthesis in the hippocampus, a key brain region for antidepressant efficacy (Adaikkan et al., 2018; Autry et al., 2011; Gideons et al., 2014; Nosyreva et al., 2013; Suzuki et al., 2017; Zanos et al., 2016). Subsequently, this signaling pathway results in the insertion of GluA1 and GluA2 subunits of the α-amino-3-hydroxy-5-methyl-4-isoxazole propionic acid receptor (AMPAR) that induces a robust synaptic potentiation of AMPAR-mediated Schaffer collateral to CA1 field excitatory postsynaptic potentials (fEPSPs) that is critical for rapid antidepressant effects (Autry et al., 2011; Nosyreva et al., 2013, 2014). While inhibition of eEF2K is necessary for the intracellular signaling required for the antidepressant and synaptic effects of ketamine, little is known about the function of eEF2K in neurons and how it regulates synaptic scaling to produce rapid antidepressant effects. A better understanding of this key synaptic substrate in mediating rapid antidepressant responses may provide new therapeutic targets for the treatment of depression.

In this study, we investigated the role of eEF2K in synaptic scaling and rapid antidepressant effects. We also performed experiments with retinoic acid (RA), which likewise regulates synaptic scaling via AMPAR protein translation in the hippocampus (Aoto et al., 2008; Poon and Chen, 2008; Sarti et al., 2012) to determine whether synaptic scaling is sufficient to produce rapid antidepressant action. We observed that synaptic scaling elicited by ketamine-eEF2K occurs independent of RA signaling and conversely, that synaptic scaling elicited by RA signaling occurs in an eEF2K-independent manner. However, these two independent signaling pathways converge at a common synaptic endpoint and produce fast-acting antidepressant-like effects.

This is an open access article under the CC BY license (http://creativecommons.org/licenses/by/4.0/).

These findings support the hypothesis that synaptic homeostatic plasticity is causally required for the behavioral effects of ketamine and represents a key substrate for rapid antidepressant action.

RESULTS

Loss of eEF2K mediates postsynaptic unsilencing in hippocampal neurons

eEF2K is a calcium/calmodulin-dependent protein kinase with one known target, eEF2. eEF2K phosphorylates eEF2, which impairs ribosomal translocation and slows the elongation phase of protein synthesis, whereas eEF2K inhibition results in desuppression of protein translation (Ryazanov et al., 1988; Suzuki and Monteggia, 2020). We initially investigated whether the loss of eEF2K alters synaptic AMPAR function in hippocampus neurons. We infected hippocampal neuronal cultures at days *in vitro* (DIV) 4 with lentivirus encoding small hairpin RNA (shRNA) (eEF2K-knockdown [KD]), which reduced endogenous eEF2K expression by ~75% and measured AMPAR-miniature excitatory postsynaptic currents (mEPSCs) at DIV 14–21 (Figures 1A and S1A–S1F). Based on previous studies examining the mechanism of action of ketamine (Autry et al., 2011; Maeng et al., 2008; Nosyreva et al., 2013, 2014), we would expect the loss of eEF2K to lead to an increase in AMPAR-mEPSCs amplitudes. Contrary to our prediction, the KD of eEF2K expression did not alter the amplitude of AMPAR-mEPSCs (Figure 1B), even in more mature neurons (DIV 18–21) (Figure S1G), but rather resulted in a significant increase in mEPSC frequency (Figures 1C and S1H). We next cultured hippocampal neurons from constitutive eEF2 knockout (eEF2K-KO) mice and recorded AMPAR-mEPSCs (Figures 1D and S1I). Similar to the eEF2K-KD experiments, the constitutive eEF2K-KO neurons did not undergo a change in mEPSC amplitudes but did display a significant increase in mEPSC frequency (Figures 1E, 1F, S1J, and S1K).

We examined the localization of eEF2 and phosphorylated-eEF2 in subcompartments of mature hippocampal neurons to understand where eEF2K acts in neurons. We found that eEF2 and its phosphorylated form were localized in dendrites and postsynaptic density protein 95 (PSD-95) positive excitatory postsynaptic spines (Figures S2 and S3A–S3E). We also identified a small inverse correlation of eEF2 phosphorylation levels with PSD-95 expression, suggesting that reduced eEF2K activity in hippocampal neurons may regulate excitatory postsynaptic function in spines via local protein synthesis of distinct targets, including possibly PSD-95 levels (Figures S3F–S3I). To examine eEF2K specifically in postsynaptic neurons, we sparsely transfected wild-type (WT) hippocampal neurons with control or the eEF2K-KD construct, which express soluble GFP to visualize and record transfected neurons that received the majority of their inputs from WT neurons (Figures 1G, S1A, S4A, and S4B). The loss of eEF2K in postsynaptic neurons did not change mEPSC amplitude, but it did significantly increase event frequency (Figures 1H–1J), revealing a cell-autonomous role for eEF2K. These observations may suggest differences in the number of excitatory synapses onto eEF2K-KD neurons. Therefore, we quantified the number of excitatory inputs onto mature hippocampal neurons transfected with eEF2K-KD compared to the control construct. However, there were no differences in the number of dendritic spines making excitatory inputs to eEF2K-KD neurons (Figures 1K and 1L). The functional changes caused by the loss of eEF2K, coupled with the lack of differences in the number of excitatory synapses, may suggest that eEF2K-KD converts silent synapses containing NMDARs but not AMPARs to those that contain both. To examine this possibility, we compared AMPA/NMDA response amplitude ratios of evoked EPSCs (eEPSC) between littermate control and eEF2K-KD neurons. AMPA/NMDA ratios was increased by eEF2K-KD, suggesting that eEF2K-KD dominantly altered the AMPAR-mediated component (Figures 1M–1P). To further confirm this finding, we sparsely transfected neurons with eEF2K-KD and recorded pharmacologically isolated AMPAR-mEPSCs and NMDAR-mEPSCs from these neurons (Figures 1Q, S4C, and S4D). The amplitudes of AMPAR-mEPSCs plotted against NMDAR-mEPSCs were similar for control and eEF2K-KD transfected neurons (Figure 1R). However, eEF2K-KD neurons showed a significant increase in AMPAR-mEPSC frequency, with no difference in NMDAR-mEPSC frequency (Figure 1S), consistent with the unmasking of postsynaptically silent synapses.

Acute blockade of NMDAR at rest or eEF2K inhibition mediates rapid synaptic scaling

In previous work, we proposed that ketamine blocks NMDARs activated under spontaneous neurotransmitter release, which is also referred to as resting neurotransmission and is independent of evoked neurotransmission, and acutely inhibits eEF2K, resulting in augmented protein synthesis of targets, including brain-derived neurotrophic factor (BDNF) and AMPARs, and potentiates synaptic responses (Autry et al., 2011; Gideons et al., 2014; Nosyreva et al., 2013; Suzuki et al., 2017). It has been shown that spontaneous NMDAR activity couples with eEF2K signaling to maintain synaptic homeostasis (Reese and Kavalali, 2015; Sutton et al., 2006, 2007). Therefore, we investigated whether eEF2K plays a role in synaptic homeostasis. Resting NMDAR activity was suppressed by co-application of the NMDAR selective competitive antagonist (2R)-amino-5-phosphonovaleric acid (AP-5) in the presence of tetrodotoxin (TTX), which inhibits the firing of action potentials, for 3 h in control or eEF2K-KD hippocampal neurons, and AMPAR-mEPSC events were recorded (Figure 2A). The TTX/AP-5 treatment significantly increased AMPAR-mEPSC amplitudes in control neurons, consistent with earlier findings (Reese and Kavalali, 2015; Sutton et al., 2006). In contrast, TTX/AP-5 did not increase AMPA-mEPSC amplitudes in eEF2K-KD neurons (Figure 2B). To determine differences in AMPAR-mEPSC amplitudes following AP-5 treatment, AMPAR-mEPSC amplitudes were randomly selected, sorted from smallest to largest, and then assembled into a rank order plot. This plot revealed that suppression of spontaneous NMDAR activity at rest scaled up synaptic weights in control neurons in a multiplicative manner (Turrigiano et al., 1998), whereas in eEF2K-KD neurons, synaptic scaling was occluded (Figure 2C). Of note, there were no significant differences of TTX/AP5 on AMPAR-mEPSC frequency with any of the conditions (Figure 2D). Similar to our findings with AP-5, ketamine also triggered synaptic scaling under resting conditions in

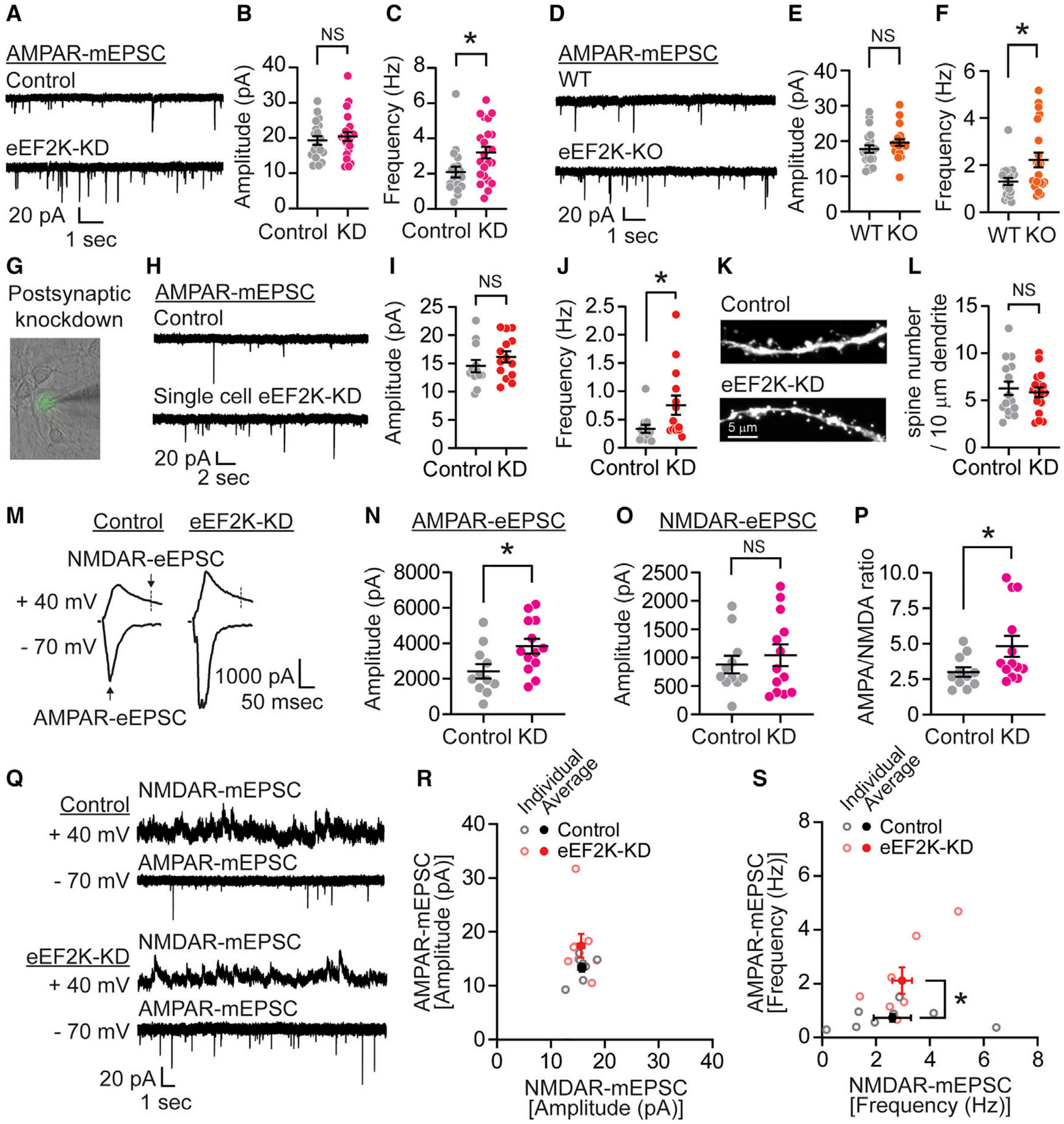

Figure 1. Loss of eEF2K leads to postsynaptic unsilencing

(A) Representative traces of AMPAR-mEPSCs from cultured hippocampal neurons infected with control or eEF2K knockdown (eEF2K-KD) lentivirus at DIV 14–21.

(B) AMPAR-mEPSC amplitude was similar between control and eEF2K-KD neurons (unpaired t test, p = 0.5303, n = 19 in control and n = 23 in eEF2K-KD).

(C) eEF2K-KD significantly increased AMPAR-mEPSC event frequency (unpaired t test, p = 0.0203, n = 19 in control and n = 23 in eEF2K-KD).

(D) Representative AMPAR-mEPSC traces from WT and eEF2K-KO cultured hippocampal neurons.

(E) AMPAR-mEPSC amplitude was similar between WT and eEF2K-KO neurons (unpaired t test, p = 0.2014, n = 21 in WT and eEF2K-KO).

(F) eEF2K-KO neurons significantly increased AMPAR-mEPSC event frequency compared to WT neurons (unpaired t test, p = 0.0114, n = 21 in WT and eEF2K-KO neurons).

(G) Experimental design to test the potential postsynaptic effects of eEF2K-KD. AMPAR-mEPSCs were recorded from single-cell eEF2K-KD neurons achieved through very low transfection efficiency.

(H) Representative traces of AMPAR-mEPSCs from cultured hippocampal neurons sparsely transfected with a control or eEF2K-KD.

(I) AMPAR-mEPSC amplitude was similar between single-cell control neurons and eEF2K-KD (unpaired t test, p = 0.2886, n = 12 in control cells and n = 14 in eEF2K-KD cells).

(J) Single-cell eEF2K-KD significantly increased AMPAR-mEPSC event frequency compared to control (unpaired t test, p = 0.0436, n = 12 in control and n = 14 in eEF2K-KD).

(K) Representative images of dendrites from cultured hippocampal neurons transfected with control or eEF2K-KD construct. Scale bar, 5 μm.

(L) Dendritic spine density was similar between eEF2K-KD neurons and control (unpaired t test, p = 0.6362, n = 16 in control and n = 17 in eEF2K-KD).

(M) Representative traces of evoked EPSC (eEPSC) at −70 mV and +40 mV in lentiviral control and eEF2K-KD neurons.

(legend continued on next page)

control neurons, whereas synaptic scaling was occluded in eEF2K-KD neurons (Figures 2E–2H). These findings are consistent with previous work demonstrating that the constitutive deletion of eEF2K impairs the induction of homeostatic synaptic scaling (Reese and Kavalali, 2015).

Our data so far shows that constitutive deletion of eEF2K results in the unmasking of postsynaptic silent synapses and impairments in the induction of homeostatic scaling. However, ketamine treatment does not result in the constitutive loss of eEF2K, but rather its acute inhibition. To more closely mimic the effect of ketamine on eEF2K inhibition (Autry et al., 2011; Gideons et al., 2014; Nosyreva et al., 2013), we acutely inhibited eEF2K activity with the selective eEF2K inhibitor A-484954 for 1 h in mature hippocampal cultured neurons and recorded AMPAR-mEPSC events (Figures 2I and S5A–S5C). Acute pharmacological inhibition of eEF2K significantly increased AMPAR-mEPSC amplitudes, triggering rapid synaptic scaling without affecting AMPAR-mEPSC frequency (Figures 2J–2L and S5D–S5G). These data show that acute suppression of eEF2K, either indirectly through treatment with an NMDA receptor antagonist or directly with the selective eEF2K inhibitor, affects excitatory synaptic function and mediates homeostatic synaptic scaling in hippocampal neurons. These findings also show that acute suppression of eEF2K elicits a different form of synaptic plasticity than chronic loss of eEF2K. Chronic impairment of eEF2K expression occludes synaptic scaling and mediates unsilencing of postsynaptic silent synapses. This result is in line with our earlier finding that constitutive loss of eEF2K impairs the rapid antidepressant responses of ketamine (Nosyreva et al., 2013). In contrast, acute eEF2K inhibition, which is relevant for the rapid antidepressant effects of ketamine (Autry et al., 2011; Gideons et al., 2014; Nosyreva et al., 2013), triggers synaptic scaling.

eEF2K and RA signaling act in an independent manner, but mediate a similar form of synaptic scaling

A similar form of synaptic scaling is mediated by RA and its receptor RA receptor α (RARα) (Aoto et al., 2008; Maghsoodi et al., 2008; Sarti et al., 2012). While RARα is a well-characterized nuclear receptor, it is also a cytoplasmic receptor, which when activated by RA desuppresses the protein synthesis of AMPAR in mature hippocampal neurons to trigger synaptic scaling (Aoto et al., 2008; Poon and Chen, 2008; Sarti et al., 2012). We examined whether eEF2K and RA act in a parallel signaling pathway to mediate synaptic scaling. We evaluated whether eEF2K is essential for RA-mediated homeostatic synaptic plasticity in hippocampal neurons. We tested the effect of direct exogenous RA application for 1 h in cultured hippocampal neurons expressing the eEF2K-KD construct (Figure 3A). RA treatment increased AMPA-mEPSC amplitudes with no effect on their frequency in control neurons, consistent with previous findings (Figures 3B and 3D) (Aoto et al., 2008; Sarti et al., 2012). Acute RA application also produced synaptic scaling in eEF2-KD neurons. The rank order plot for AMPA-mEPSC amplitudes of eEF2K-KD neurons was similar to control neurons, suggesting that RA-mediated synaptic scaling is independent of eEF2K (Figures 3B and 3C). As a complementary approach, we cultured hippocampal neurons from floxed RARα (RARα$^{fl/fl}$) mice, infected them with lentivirus expressing GFP-tagged Cre recombinase or GFP alone, and acutely treated them with A-484954 (Figure 3E). RARα mRNA expression was significantly reduced ~90% in RARα-KO culture (Figure S5H). Acute pharmacological inhibition of eEF2K induced similar synaptic scaling in GFP-infected control and RARα-KO neurons (Figures 3F–3H), indicating that synaptic scaling triggered by acute eEF2 inhibition does not require RARα. Our earlier observation that chronic loss of eEF2K unmasks silent synapses could involve a contribution of RARα signaling. We therefore expressed eEF2K-KD in cultured RARα-KO neurons and recorded AMPA-mEPSC events. We observed an increase in AMPA-mEPSC frequency similar to eEF2K-KD in control neurons (Figures S5I–S5K), showing that chronic loss of eEF2K-mediated synapse unsilencing is also RARα independent. These results show that acute eEF2K inhibition mediates rapid synaptic scaling independent of RA signaling and vice versa. Furthermore, RA treatment did not induce further scaling after eEF2K inhibition-mediated synaptic scaling, suggesting that these signaling pathways target the same functional synaptic scaling endpoint (Figures 3I–3L). These results suggest that signaling pathways driven by acute eEF2K inhibition and RA act in parallel but converge at the final synaptic endpoint to mediate homeostatic synaptic scaling.

RARα is not essential in ketamine-mediated fast-acting antidepressant action, but direct RARα activation triggers rapid antidepressant action

We next examined whether the parallel nature of the eEF2K and RA pathways also applied to their ability to drive ketamine-mediated homeostatic synaptic potentiation in acute hippocampal slices. Previous findings revealed rather surprisingly that ketamine produces a robust synaptic potentiation in Schaffer collateral to CA1 fEPSPs of WT mice (Autry et al., 2011) that is eEF2K dependent, as this synaptic potentiation is impaired in eEF2K-KO mice (Nosyreva et al., 2013). To examine the involvement

(N) AMPAR-eEPSC was significantly increased by eEF2K-KD (unpaired t test, $p = 0.0246$, $n = 11$ in control and $n = 13$ in eEF2K-KD).
(O) NMDAR-eEPSC of eEF2K-KD was similar to that of control (unpaired t test, $p = 0.5224$, $n = 11$ in control and $n = 13$ in eEF2K-KD).
(P) AMPA/NMDA ratio was significantly increased by eEF2K-KD (unpaired t test, $p = 0.0477$, $n = 11$ in control and $n = 13$ in eEF2K-KD).
(Q) Representative AMPAR-mEPSC and NMDAR-mEPSC traces from neurons sparsely transfected with control or eEF2K-KD construct.
(R) AMPAR-mEPSC amplitudes were plotted against NMDAR-mEPSC amplitudes in scatterplot (control and eEF2K-KD individual values: gray and light red circles, respectively; control and eEF2K-KD average values: black and red closed circles, respectively). AMPAR-mEPSC and NMDAR-mEPSC amplitudes were similar between eEF2K-KD and control neurons (unpaired t test, AMPAR-mEPSC $p = 0.1070$, NMDAR-mEPSC $p = 0.8466$, $n = 8$ in control and eEF2K-KD).
(S) Same as the amplitude, AMPAR-mEPSC frequencies were plotted against NMDAR-mEPSC frequencies in scatterplot. eEF2K-KD neurons significantly increased AMPAR-mEPSC frequency but not NMDAR-mEPSC frequency compared to control neurons (unpaired t test, AMPAR-mEPSC $p = 0.0183$, NMDA-mEPSC $p = 0.6517$, $n = 8$ in control and eEF2K-KD).
Data are represented as means ± SEMs.
See also Figures S1–S4.

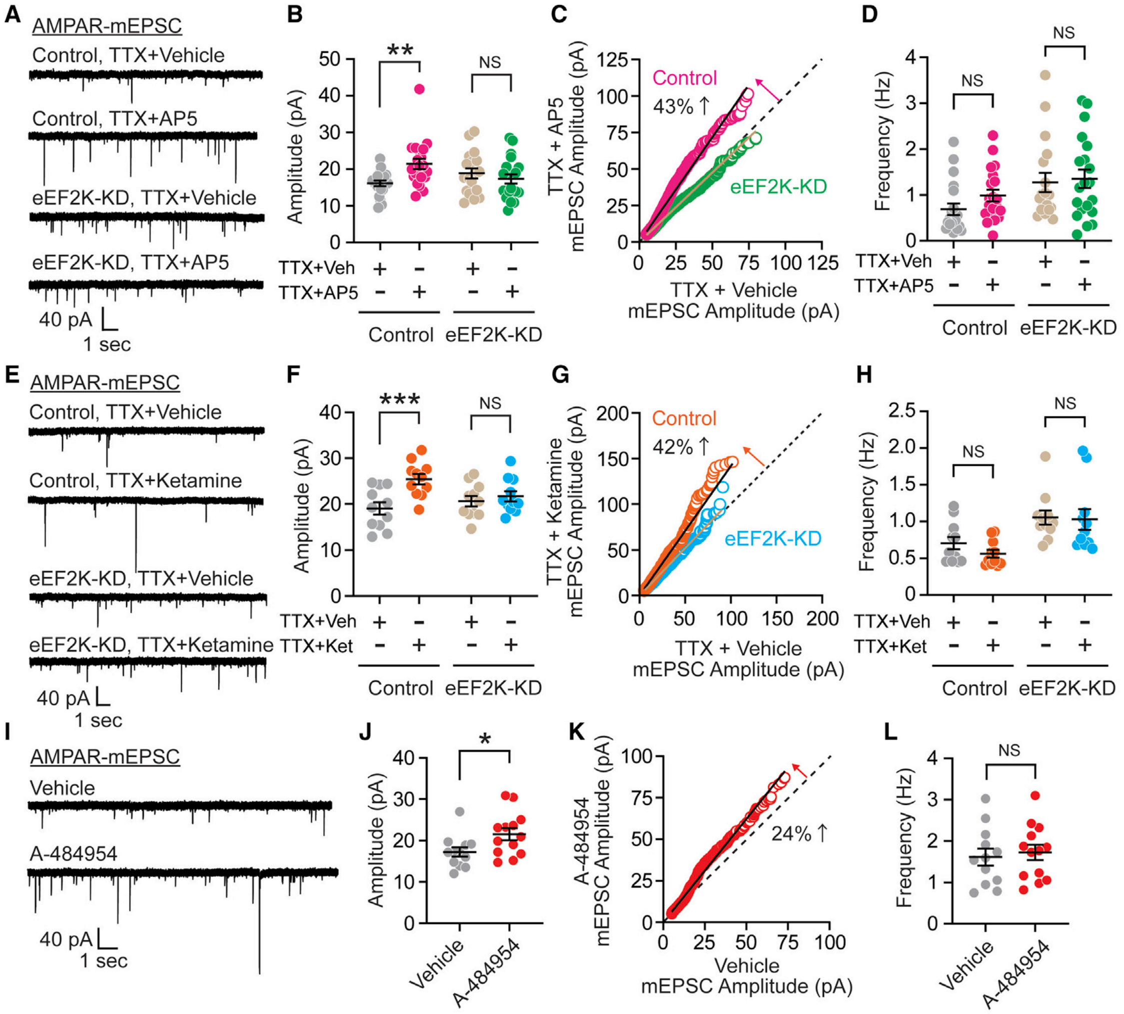

Figure 2. Acute inhibition of eEF2K activity induces rapid synaptic scaling

(A) Representative traces of AMPAR-mEPSC from lentiviral control or eEF2K-KD cultured hippocampal neurons treated with or without 50 μM AP-5 in the presence of 1 μM TTX for 3 h.

(B) TTX/AP-5 treatment significantly increased AMPAR-mEPSC amplitude in control neurons, but this increase was occluded in eEF2K-KD neurons (2-way ANOVA with Sidak's multiple comparisons, TTX + vehicle versus TTX + AP-5 in control p = 0.0053, TTX + vehicle versus TTX + AP-5 in eEF2K-KD p = 0.6257, n = 18–20 per group).

(C) Rank order plot showed a 43% increase in synaptic strength in control neurons treated with TTX/AP-5 (linear regression, slope = 1.43). Conversely, rank order plot did not show a multiplicative increase in synaptic strength in eEF2K-KD neurons treated with TTX/AP-5 (linear regression, slope = 0.95).

(D) AMPAR-mEPSC frequency was not altered by TTX/AP-5 treatment in the control and eEF2K-KD neurons (2-way ANOVA with Sidak's multiple comparisons, TTX + vehicle versus TTX + AP-5 in control p = 0.3667, TTX + vehicle versus TTX + AP-5 in eEF2K-KD p = 0.9349, n = 18–20 per group).

(E) Representative traces of AMPAR-mEPSC from lentiviral control or eEF2K-KD cultured hippocampal neurons treated with or without 50 μM ketamine in the presence of 1 μM TTX for 3 h.

(F) Similar to AP-5, ketamine treatment significantly increased AMPAR-mEPSC amplitude in control neurons in the presence of TTX, but this increase was occluded in eEF2K-KD neurons (2-way ANOVA with Sidak's multiple comparisons, TTX + vehicle versus TTX + ketamine in control p = 0.0009, TTX + vehicle versus TTX + ketamine in eEF2K-KD p = 0.7840, n = 11 per group).

(G) Rank order plot showed a 42% increase in synaptic strength in control neurons treated with TTX/ketamine (linear regression, slope = 1.42). Conversely, rank order plot did not show a multiplicative increase in synaptic strength in eEF2K-KD neurons treated with TTX/ketamine (linear regression, slope = 1.04).

(H) AMPAR-mEPSC frequency was not altered by TTX/ketamine treatment in the control and eEF2K-KD neurons (2-way ANOVA with Sidak's multiple comparisons, TTX + vehicle versus TTX + ketamine in control p = 0.5344, TTX + vehicle versus TTX + ketamine in eEF2K-KD p = 0.9780, n = 11 per group).

(I) Representative traces of AMPAR-mEPSC from WT cultured hippocampal neurons with treatment of vehicle or 30 μM A-484954 for 1 h.

(J) Acute treatment of A-484954 significantly increased AMPAR-mEPSC amplitude (unpaired t test, p = 0.0292, n = 12 in vehicle-treated neurons and n = 13 in A-484954-treated neurons).

(legend continued on next page)

of the RA pathway, we assessed the effect of ketamine on hippocampal fEPSPs in the CA1 region of RARα-KO mice. While the inhibition of spontaneous NMDAR activity can promote RA synthesis (Aoto et al., 2008; Wang et al., 2011), we nevertheless observed that ketamine application potentiated evoked fEPSPs in hippocampal slices from RAR-KOs similar to that observed in WT control mice (Figure 4A), indicating that RA signaling through RARα is not required for the ketamine-mediated synaptic potentiation.

Given our focus on antidepressant action, we next examined whether ketamine requires RARα signaling to produce its behavioral effects. We administered a single low dose of ketamine to RARα-KOs and tested the mice in the forced swim test (FST). Ketamine significantly reduced immobility in the RARα-KO mice similar to littermate controls, suggestive of an antidepressant-like effect (Figure 4B) and showing that RA signaling is not required for the antidepressant action of ketamine. These findings, as well as data showing acute eEF2K inhibition mediates homeostatic plasticity in an independent pathway from RA signaling, provide a unique opportunity to directly investigate a role for homeostatic synaptic scaling in rapid antidepressant action. To determine whether homeostatic synaptic potentiation can elicit rapid antidepressant effects, we performed electrophysiological and behavioral experiments using a RARα analog. The selective RARα agonist AM580 induced synaptic scaling in cultured hippocampal neurons and triggered synaptic potentiation in hippocampal slices (Figures 4C and 4D and S6A–S6D). Notably, this synaptic potentiation was occluded by ketamine-induced synaptic potentiation, further suggesting that RARα activation triggers a similar form of homeostatic synaptic potentiation with ketamine (Figures 4C and 4D). We next acutely treated C57BL/6 mice with AM580 and tested them in the FST or novelty-suppressed feeding (NSF) test. A single administration of AM580 triggered rapid antidepressant-like responses in mice in these two behavioral paradigms (Figures 4E, S6E, and S6F). Co-treatment of mice with AM580 and NBQX, an AMPAR antagonist, abolished the antidepressant effects and demonstrated a requirement of AMPAR activation in the behavioral response (Figure 4F). The sensitivity of antidepressant action to AMPAR block is consistent with previous ketamine studies (Autry et al., 2011; Maeng et al., 2008; Nosyreva et al., 2013) and indicates that both AM580 and ketamine rely on AMPAR-mediated synaptic plasticity to exert their behavioral effects. AM580 also produced a sustained antidepressant-like response up to 7 days postinjection similar to ketamine (Figures 4G, S6G, and S6H). We also exposed mice to chronic restraint stress (CRS) to more closely mimic an animal model of depression and then tested them in the sucrose preference test (SPT). We observed that AM580 produced an antidepressant-like response compared to vehicle treatment in chronically stressed animals (Figure 4H). While BDNF is a key mediator for the rapid antidepressant action of ketamine (Autry et al., 2011; Gideons et al., 2014; Nosyreva et al., 2013), AM580 did not alter BDNF protein expression in the hippocampus, further highlighting the distinct signaling mechanism between these two pathways (Figures S6I and S6J). We did confirm an increase in the phosphorylation level of extracellular signal-regulated kinase 1/2 (ERK1/2) as a downstream signaling of RARα activation (Figures S6I and S6K) (Chen and Napoli, 2008). These data show that NMDAR-eEF2K and RA signaling induce homeostatic synaptic scaling and behavioral antidepressant-like response via independent signaling pathways.

Multiplicative scaling is triggered by the RARα agonist or ketamine in stratum radiatum of hippocampus CA1 region

We next directly examined whether the RARα agonist and ketamine trigger homeostatic synaptic scaling *in vivo*. For this purpose, we used immunolabeling of surface AMPARs to determine the mechanisms underlying homeostatic plasticity in rapid antidepressant action. We acutely treated C57BL/6 mice with AM580 and performed immunohistochemistry using antibodies against the extracellular domain of GluA1 and GluA2 to detect surface AMPAR subunits in stratum radiatum of hippocampus CA1 (Figures 5A and 5B). Surface GluA1 and GluA2 were detected by confocal microscopy and analyzed using particle analysis after applying watershed segmentation (Figure S7A). The histogram of GluA1 puncta size showed a similar distribution between vehicle and AM580 treatment, suggesting that GluA1 puncta size was not altered by AM580 (Figure 5C). The fluorescence distributions could be fitted with a Gaussian function, suggesting that selected puncta fluorescence values within the Gaussian curve correspond to single postsynaptic GluA1 clusters. In contrast, large puncta outside these fits may be attributed to fluorescence values originating from multiple adjacent postsynaptic sites. To assess the effect of AM580 on GluA1 intensities, we ranked total intensities of GluA1 puncta, which are distributed in a Gaussian peak in area analysis, and plotted rank order plots similar to the electrophysiological analysis and observed increased GluA1 intensities by AM580 treatment in a multiplicative manner (Figure 5D). We analyzed GluA2 puncta area and intensities using the same strategy. However, the slope in rank order plot was close to 1, indicating that RARα activation does not increase GluA2 (Figures 5E and 5F). The finding that RA signaling increases only GluA1 subunits postsynaptically is consistent with previous reports that RARα activation induces synaptic scaling through an insertion of homomeric GluA1-containing AMPARs (Aoto et al., 2008; Wang et al., 2011). We also treated mice with ketamine and quantified the surface GluA1 and GluA2 puncta in stratum radiatum of hippocampus CA1. We observed a similar distribution of both GluA1 and GluA2 puncta size, but the slopes in rank order plot for those puncta intensities were increased by the treatment of ketamine. These data agree with previous findings that both GluA1 and GluA2

(K) Rank order plot indicates 24% increase in synaptic strength after acute eEF2K inhibition (linear regression, slope = 1.24).
(L) AMPAR-mEPSC frequency was not changed by A-484954 treatment (unpaired t test, p = 0.6885, n = 12 in vehicle-treated neurons and n = 13 in A-48495-treated neurons).
Data are represented as means ± SEMs.
See also Figure S5.

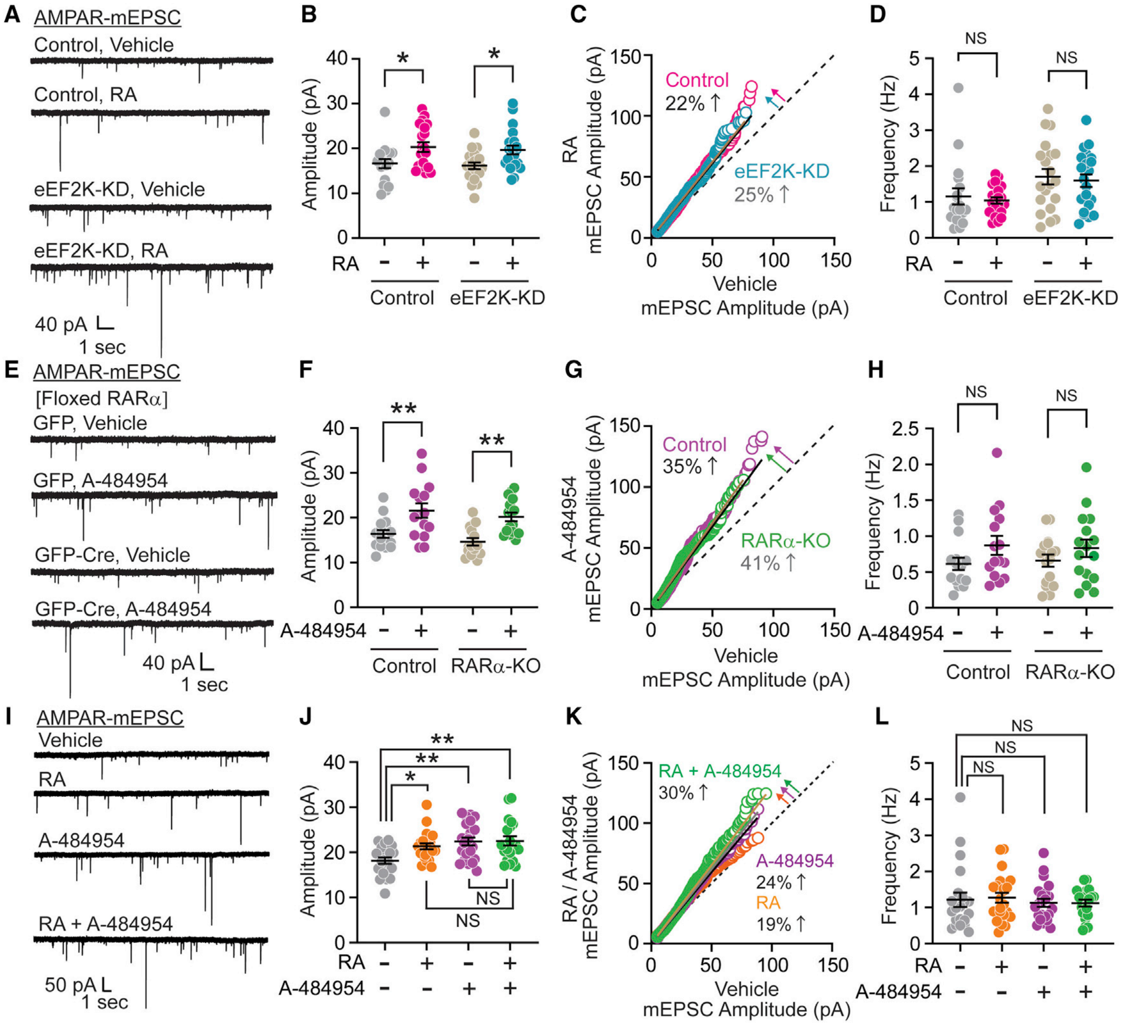

Figure 3. eEF2K and RA signaling act in an independent manner, but mediate similar forms of synaptic scaling

(A) Representative traces of AMPAR-mEPSC from lentiviral control or eEF2K-KD cultured hippocampal neurons with treatment of vehicle or 1 μM RA for 1 h.

(B) Acute treatment of RA induced synaptic scaling in both control and eEF2K-KD neurons (2-way ANOVA with Sidak's multiple comparisons, vehicle versus RA in control p = 0.0205, vehicle versus RA in eEF2K-KD p = 0.0203, n = 18–21 per group).

(C) Rank order plot showed a 22% increase in synaptic strength after acute RA treatment in control neurons (linear regression, slope = 1.22). Similar to control neurons, eEF2K-KD neurons also showed a 25% upscaling after acute RA treatment (linear regression, slope = 1.25).

(D) AMPAR-mEPSC frequency was not altered by RA treatment in control or eEF2K-KD neurons (2-way ANOVA with Sidak's multiple comparisons, vehicle versus RA in control p = 0.8931, vehicle versus RA in eEF2K-KD p = 0.8898, n = 18–21 per group).

(E) Representative traces of AMPAR-mEPSC from control or RARα-KO cultured hippocampal neurons with treatment of vehicle or 30 μM A-484954 for 1 h. RARα-KO cultured neurons were generated by Cre expression in RARα$^{fl/fl}$ hippocampal cultures.

(F) Acute treatment of A-484954 significantly enhanced AMPAR-mEPSC amplitude in control and RARα-KO neurons (2-way ANOVA with Sidak's multiple comparisons, vehicle versus A-484954 in control p = 0.0027, vehicle versus A-484954 in RARα-KO p = 0.0012, n = 15–16 per group).

(G) Rank order plot for AMPAR-mEPSC amplitude with or without A-484954 treatment in control and RARα-KO neurons (linear regression, slope = 1.35 in control and = 1.41 in RARα-KO).

(H) Acute treatment of A-484954 did not significantly change AMPAR-mEPSC frequency (2-way ANOVA with Sidak's multiple comparisons, vehicle versus A-484954 in control p = 0.1801, vehicle versus A-484954 in RARα-KO p = 0.4409, n = 15–16 per group).

(I) Representative traces of AMPAR-mEPSC from WT cultured hippocampal neurons treated with vehicle, 1 μM RA, 30 μM A-484954, or 1 μM RA with 30 μM A-484954 for 1 h.

(J) Acute treatment of A-484954 or RA significantly increased AMPAR-mEPSC amplitude. Co-treatment of RA with A-484954 also increased AMPA-mEPSC amplitude, but it was similar to that in the treatment of A484954 or RA (1-way ANOVA with Tukey's multiple comparisons, vehicle versus RA p = 0.0345, vehicle versus A-484954 p = 0.0022, vehicle versus A-484954 + RA p = 0.0022, RA versus RA + A-484954 p = 0.7471, A-484954 versus RA + A484954 p = 0.9998, n = 20–22 per group).

(legend continued on next page)

subunits are required for the synaptic potentiation and antidepressant effects of ketamine (Nosyreva et al., 2013). As a control to further validate our methodology, we pretreated mice with the protein synthesis inhibitor anisomycin before ketamine treatment as the rapid antidepressant action of ketamine is dependent on protein translation (Figures 5G and 5H) (Autry et al., 2011; Nosyreva et al., 2013). Anisomycin pretreatment before ketamine reduced the slope in rank order plot compared to ketamine alone, indicating that new protein synthesis of GluA1 and GluA2 is required for ketamine-mediated synaptic scaling (Figures 5I–5L). These findings demonstrate that the RARα agonist AM580 as well as ketamine alter synaptic weight multiplicatively in the CA1 at doses that trigger rapid antidepressant-like effects. These findings also reveal that the RARα agonist and ketamine mediate synaptic scaling in the hippocampus through different AMPAR-mediated mechanisms.

DISCUSSION

In this study, we establish a causal link between multiplicative synaptic upscaling and fast-acting antidepressant effects and propose that this form of synaptic plasticity is a major synaptic substrate required for the efficacy of rapid antidepressant action. Multiplicative synaptic upscaling is elicited in response to sustained—hours to days—block of neuronal activity and/or synaptic inputs and results in an increase in synaptic strength via the postsynaptic insertion of AMPARs in quantities that are proportional to the amount of preexisting AMPAR clusters in a synapse. In this way, synaptic upscaling is expected to preserve the relative strengths of synapses to not disrupt information storage while enabling global control of synaptic activity (Kavalali and Monteggia, 2020; Turrigiano et al., 1998, 2008). When considered individually, ketamine- and the RA-mediated signaling are both associated with this form of plasticity. Notably, these signaling pathways both enhance synaptic strength rapidly via dendritic protein translation. Here, we demonstrate that these two signaling pathways act independently to converge on the singular target of synaptic upscaling to mediate the behavioral effects (Figure 6). Synaptic scaling triggered by ketamine or RA is abrogated by the genetic or pharmacological impairment of the signaling pathways, which in turn suppresses the behavioral response. In the literature, the question of causality is often addressed with optogenetic or chemogenetic experiments by stimulating or silencing particular sets of neurons and then linking them to a particular behavioral response. While these manipulations can give rise to homeostatic forms of plasticity (Rogers et al., 2021), their ability to elicit multiplicative synaptic scaling remains to be validated. Therefore, here, to establish causality, we took advantage of complementary pharmacological and genetic approaches to probe the intersection of ketamine- and RA-mediated signaling pathways that unequivocally trigger "multiplicative synaptic scaling" independently while eliciting the same antidepressant-like behaviors.

To elucidate the link between ketamine-induced intracellular signaling and synaptic scaling, we examined the chronic loss of function as well as the acute inhibition of eEF2K to address the specific role of eEF2K in ketamine-mediated rapid antidepressant action. Rather unexpectedly, we found that the chronic loss of eEF2K signaling elicits postsynaptic unsilencing. Silent synapses are considered as immature excitatory glutamatergic synapses and represent the majority of synapses at early developmental stages in the brain. During postnatal development, silent synapses are converted into functional synapses containing AMPARs that are recruited into the postsynaptic membrane (Hanse et al., 2013; Kerchner and Nicoll, 2008). Previous work has reported that basal NMDAR activity suppresses the insertion of AMPARs into silent synapses in the hippocampus (Adesnik et al., 2008). In agreement with this premise, our results suggest that basal NMDAR-mediated neurotransmission-driven eEF2K activity, via Ca^{2+}/CaM, leads to slow local protein translation and suppresses synaptic unsilencing. However, further studies will be necessary to assess this hypothesis since chronic regulation of NMDAR function is complex on synaptic function (Liao et al., 1999; Rao and Craig, 1997).

In contrast to constitutive eEF2K loss, acute inhibition of eEF2K activity mediates synaptic scaling. Our current findings agree with previous work showing that protein translation, in particular synthesis of new AMPARs, is a critical mechanism to induce synaptic scaling downstream of acute resting NMDAR activity block or transient eEF2K inhibition (Autry et al., 2011; Nosyreva et al., 2013; Reese and Kavalali, 2015; Sutton et al., 2006, 2007). Thus, transient eEF2K inhibition leads to the recruitment of AMPARs within the postsynaptic specializations that results in the induction of synaptic upscaling. We show that eEF2K is localized in postsynaptic regions of excitatory neurons and triggers distinct synaptic effects following constitutive loss compared to acute/transient inhibition. Constitutive loss of eEF2K signaling elicits postsynaptic unsilencing and occludes the rapid antidepressant effects of ketamine (Nosyreva et al., 2013). In contrast, acute eEF2K inhibition, which can be elicited by ketamine-mediated NMDAR block, decreases eEF2 phosphorylation, augments local protein synthesis, and produces synaptic potentiation that drives the rapid antidepressant effects (Autry et al., 2011).

RA signaling has been suggested to play a role in cognition, neurological, and psychiatric disorders (Lane and Bailey, 2005; Wołoszynowska-Fraser et al., 2020). A vitamin A derivative, isotretinoin (13-cis-retinoic acid), is prescribed for the treatment of acne. While there are reports suggesting that isotretinoin may increase the risk of depression and suicide, a possible link between isotretinoin and depression remains equivocal, as other studies failed to demonstrate an association with depression

(K) Rank order plot for AMPAR-mEPSC amplitude with drug treatment compared with vehicle treatment (linear regression, slope = 1.19 in RA, = 1.24 in A-484954, = 1.30 in RA+A-484954).

(L) AMPAR-mEPSC frequencies in all drug treatment conditions were similar to that in vehicle treatment (1-way ANOVA with Tukey's multiple comparisons, vehicle versus RA p = 0.9909, vehicle versus A-484954 p = 0.9770, vehicle versus RA + A-484954 p = 0.9716, n = 20–22 per group).

Data are represented as means ± SEMs.

See also Figure S5.

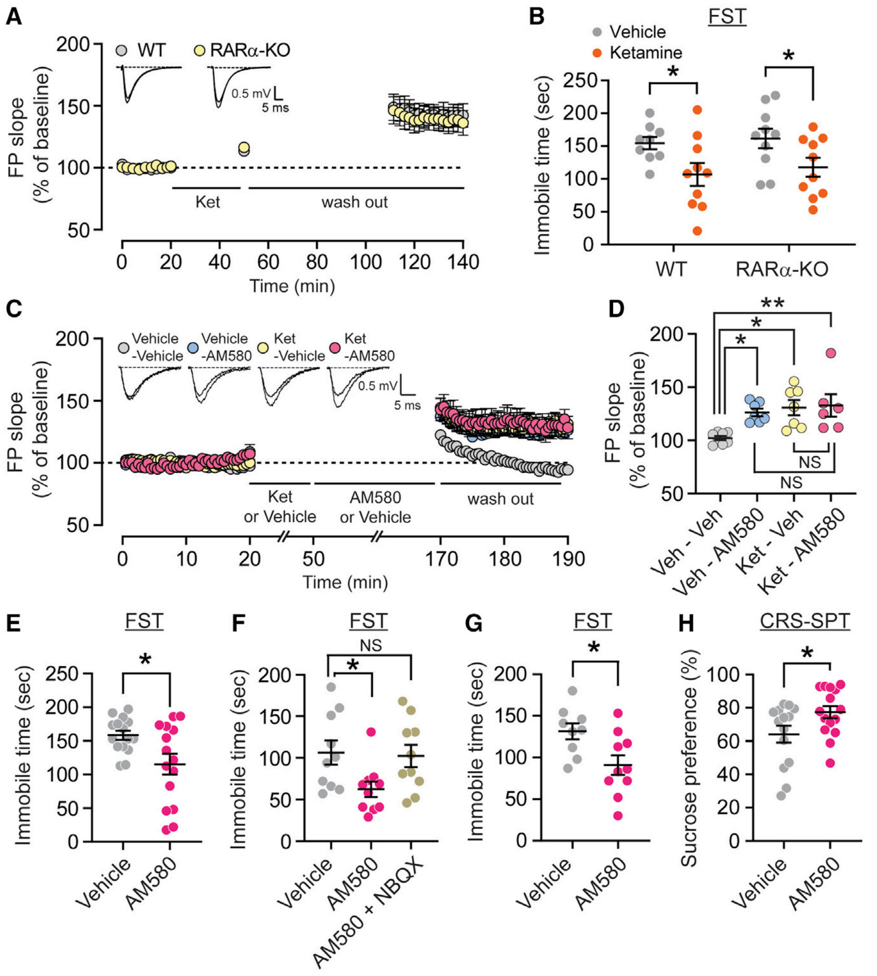

Figure 4. RARα is not essential in ketamine-mediated fast-acting antidepressant action but direct RARα activation triggers behavioral rapid antidepressant effect

(A) Field excitatory postsynaptic potentials (fEPSPs) in 20 μM ketamine-treated WT and RARα-KO hippocampal slices. Initial field potential (FP) slopes was plotted as a function of time. Inset, representative wave forms from WT and RARα-KO slices before and after treatment.

(B) RARα-KO mice showed rapid antidepressant effect of ketamine in the forced swim test (FST) 30 min after injection (2-way ANOVA with Fisher's least significant difference [LSD] test, vehicle versus ketamine in WT p = 0.0287, vehicle versus ketamine in RARα-KO p = 0.0377, n = 9–10 per group).

(C) fEPSPs were recorded from WT hippocampal slice treated with AM580 in the presence or absence of ketamine pretreatment. After 20 min of stable baseline measurement, 20 μM ketamine or vehicle were applied for 30 min. After these pretreatments, 20 μM AM580 was applied for 2 h and then fEPSP responses were recorded for 20 min. Inset, representative wave forms before and after treatment.

(D) AM580-mediated synaptic potentiation did not show further potentiation in the presence of ketamine pretreatment (1-way ANOVA with Tukey's multiple comparisons, vehicle-vehicle versus vehicle-AM580 p = 0.0362, vehicle-vehicle versus ketamine-vehicle p = 0.0107, vehicle-vehicle versus ketamine-AM580 p = 0.0086, vehicle-AM580 versus ketamine-AM580 p = 0.8866, ketamine-vehicle versus ketamine-AM580 p = 0.9958, n = 6–8 per group).

(E) Immobile time of mice treated with AM580 at 20 mg/kg in FST 2 h after injection (unpaired t test, p = 0.0164, n = 15 per group).

(F) Immobility of mice administrated vehicle, AM580 (20 mg/kg), or AM580 + NBQX (10 mg/kg) in FST 2 h postinjection (1-way ANOVA with Dunnett's multiple comparisons, vehicle versus AM580 p = 0.0367, vehicle versus AM580 + NBQX p = 0.9619, n = 10 per group).

(G) AM580 (20 mg/kg) significantly reduced an immobility in FST at 7 days after injection (unpaired t test, p = 0.0182, n = 9 in vehicle and n = 10 in AM580).

(H) Sucrose preference of chronic restraint stressed mice treated with AM580 at 20 mg/kg in sucrose preference test (SPT) (unpaired t test, p = 0.0413, n = 14 in vehicle and n = 15 in AM580).

Data are represented as means ± SEMs.

See also Figure S6.

or rather showed the amelioration of depressive symptoms (Huang and Cheng, 2017; Jacobs et al., 2001; Jick et al., 2000; Li et al., 2019; Magin et al., 2005). Moreover, the mechanism underlying the potential effects of isotretinoin in the brain remain unexplored. While the role of RA signaling in depression is unclear, a potential link to antidepressant action has not been investigated. An understanding of the basic synaptic mechanisms associated with RA action in the brain is needed to place its therapeutic potential and side effects into context. Previous studies have reported a function of RARα as a regulator for AMPAR translation and homeostatic plasticity in hippocampal neurons (Aoto et al., 2008; Sarti et al., 2012; Wang et al., 2011). Here, we demonstrate that the acute activation of RARα triggers synaptic scaling in hippocampal neurons and mediates antidepressant-like effects in mice.

Our results also show that RA signaling acting through RARα mediates a similar form of synaptic plasticity in the hippocampus as acute eEF2K inhibition, although these pathways are molecularly independent. RARα contains a mRNA-binding domain and represses the protein translation of target mRNAs, including GluA1. When RARα is activated through the binding of RA or an RARα agonist, it reduces mRNA binding and de-represses translation (Aoto et al., 2008; Poon and Chen, 2008; Sarti et al., 2012). In contrast, eEF2K and eEF2 regulate peptide elongation during protein translation, which constitutes a distinct mechanism for protein expression de-suppression compared to the RARα pathway (Suzuki and Monteggia, 2020).

Previous evidence has shown that treatment with an AMPAR antagonist blocks the antidepressant action of ketamine in preclinical models (Li et al., 2010; Maeng et al., 2008). The requirement for AMPAR activity for plasticity elicited by a NMDAR blocker was initially puzzling. However, the finding that ketamine, by blocking NMDARs, triggers protein synthesis-dependent insertion of new AMPARs and subsequent synaptic

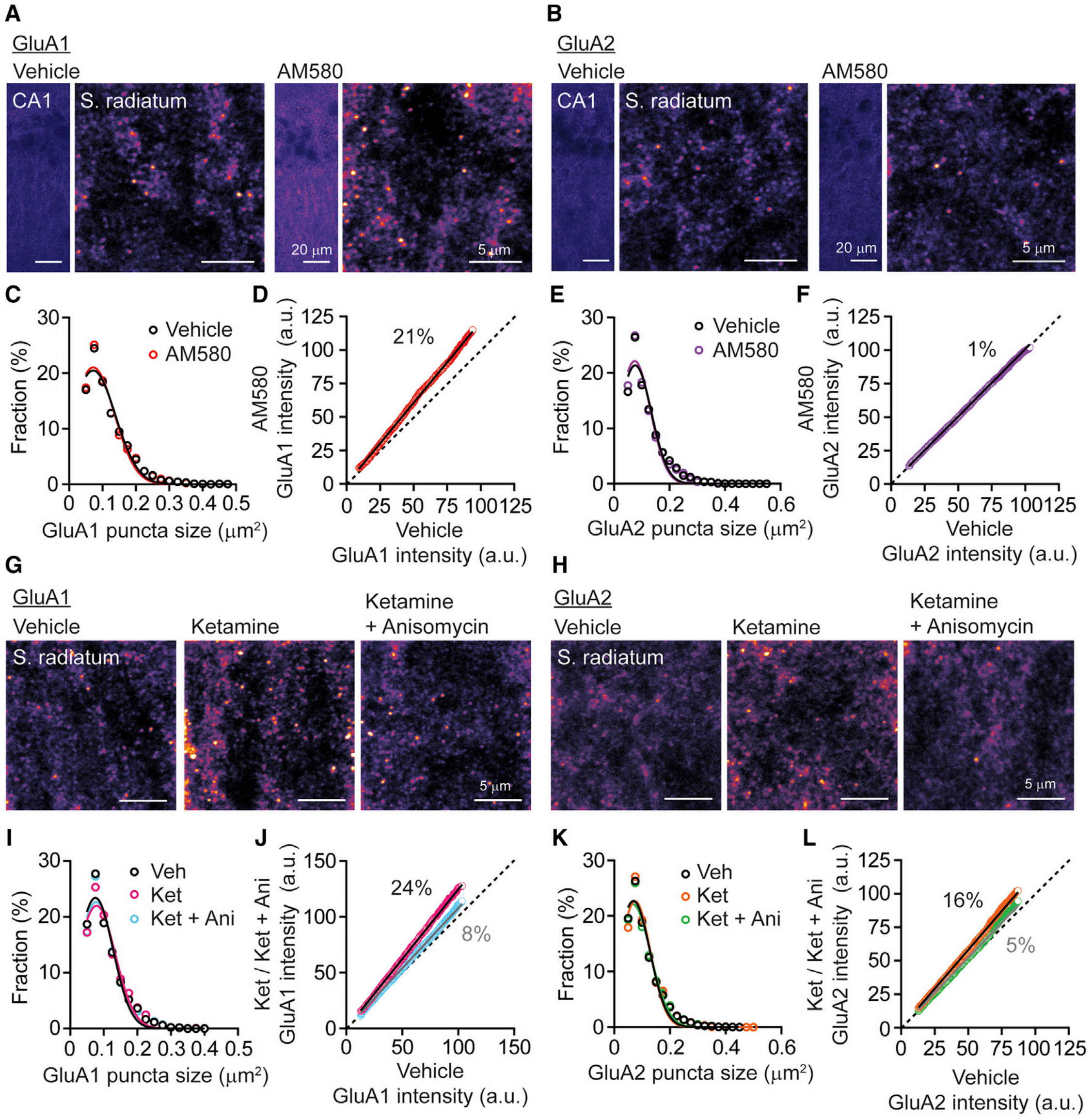

Figure 5. Multiplicative scaling is triggered by treatment of RARα agonist or ketamine in stratum radiatum of hippocampus CA1

(A and B) Representative surface GluA1 and GluA2 images of hippocampus CA1 region and stratum radiatum layers 2 h after treatment with vehicle or AM580 (20 mg/kg). Scale bar, 20 or 5 μm.

(C) Distribution of individual GluA1 puncta size (vehicle, n = 3,824; AM580, n = 3,998 from 4 mice in each condition). The histogram was fitted by Gaussian distribution (vehicle, mean + 3 SD = 0.272 μm^2; AM580, mean + 3 SD = 0.262 μm^2).

(D) Rank order plot showed an increase in surface GluA1 multiplicatively in CA1 stratum radiatum of mice treated with AM580 (linear regression, slope = 1.21, n = 3,368).

(E) Distribution of individual GluA1 puncta size (vehicle, n = 4,156; AM580, n = 4,287 from 4 mice in each condition). The histogram was fitted by Gaussian distribution (vehicle, mean + 3 SD = 0.250 μm^2; AM580, mean + 3 SD = 0.243 μm^2).

(F) Rank order plot did not show multiplicative increase in surface GluA2 in CA1 stratum radiatum of mice treated with AM580 (linear regression, slope = 1.01, n = 3,612).

(G and H) Representative surface GluA1 and GluA2 images of hippocampus CA1 region and stratum radiatum layers 2 h after treatment with vehicle or ketamine (5 mg/kg). Anisomycin (100 mg/kg) was administrated 20 min before ketamine treatment. Scale bar, 5 μm.

(I) Distribution of individual GluA1 puncta size (vehicle, n = 4192; ketamine, n = 3704; ketamine + anisomycin, n = 3,699 from 4 mice in each condition). The histogram was fitted by Gaussian distribution (vehicle, mean + 3 SD = 0.233 μm^2; ketamine, mean + 3 SD = 0.247 μm^2, ketamine + anisomycin, mean + 3 SD = 0.238 μm^2).

(J) Rank order plot showed multiplicative increase in surface GluA1 in CA1 stratum radiatum of mice treated with ketamine, but anisomycin pretreatment decreased slope (linear regression, slope = 1.24 in ketamine and = 1.08 in ketamine + anisomycin, Mann-Whitney test, $p < 0.0001$ in ketamine compared to ketamine + anisomycin, n = 3246).

(legend continued on next page)

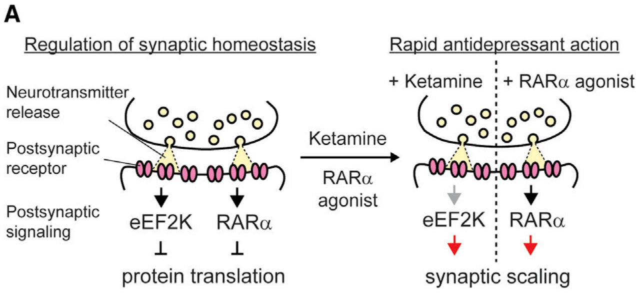

Figure 6. Emerging view on the mechanism of rapid antidepressant action

(A) eEF2K and RARα act as regulators for protein translation and synaptic homeostasis. Spontaneous synaptic neurotransmission activates eEF2K via Ca^{2+}/CaM binding, which in turn increases eEF2 phosphorylation and suppresses dendritic protein translation. In addition, synaptic activity regulates RA synthesis and RARα represses dendritic protein translation. Ketamine blocks NMDAR during spontaneous glutamatergic neurotransmitter release, which in turn acutely inhibits eEF2K activity to trigger homeostatic synaptic scaling and rapid antidepressant-like effect. However, RARα agonists induce synaptic scaling via RARα activation to exert rapid antidepressant-like effect. Ketamine and RARα agonists alter synaptic weight multiplicatively, which may be a key synaptic substrate for rapid antidepressant action.

potentiation in Schaffer collateral to CA1 pyramidal neuron synapses provided a mechanistic link. Further studies showed that AMPAR antagonists inhibit the ketamine-mediated synaptic potentiation and strengthened the link between the requirement for synaptic potentiation and antidepressant action (Autry et al., 2011; Nosyreva et al., 2013). We also found that RA signaling follows a similar sequence of events and requires AMPARs for synaptic potentiation and antidepressant-like behavioral effects. However, the contribution of specific AMPAR subunits is different between these two drugs. Our findings show that RARα activation increases GluA2-lacking GluA1 homomers in the CA1 region of the hippocampus, whereas ketamine increases GluA2-containing AMPARs. Given that AMPAR subunit compositions alter Ca^{2+} permeability and channel conductance (Cull-Candy et al., 2006; Isaac et al., 2007; Lee, 2012; Liu and Zukin, 2007), the cellular response of RARα activation and ketamine may be different. Nevertheless, these data support the notion that two parallel forms of signaling converge on a synaptic endpoint by eliciting a homeostatic form of plasticity that is required for rapid antidepressant action.

Clinical neuroimaging studies have examined potential brain regions involved in the rapid antidepressant action of ketamine, with the hippocampus identified as a key region (Abdallah et al., 2015; Ionescu et al., 2018; Lally et al., 2015). In separate work, S-ketamine has been shown to increase hippocampal volume, in particular in the CA1 region, starting at 65 min post-administration, suggesting the early engagement of the hippocampal circuitry in rapid antidepressant responses (Höflich et al., 2021). Synaptic plasticity elicited by multiplicate synaptic upscaling plays a crucial role in the stability of neuronal excitability and network activity by functionally modifying connectivity across multiple neurons and circuit dynamics (Turrigiano, 2008). Although our experiments do not address why synaptic scaling is a key substrate for rapid antidepressant action, it is tempting to speculate that the synaptic enhancement in the hippocampus that rapidly follows ketamine application or synaptic RA signaling activation may fix negative memories and emotions or enhance motivation and cognition with other brain regions such as the amygdala and nucleus accumbens. Future studies will be necessary to investigate how synaptic scaling in the hippocampus alters neuronal circuits and modulates the function of other brain regions. Here, we identified synaptic scaling in the hippocampus that is produced by either ketamine or RA and showed its impact on rapid antidepressant action. These findings demonstrate that the global augmentation of synaptic efficacy via synaptic upscaling in the hippocampus is causally linked to the behavioral effects of ketamine. This form of homeostatic synaptic plasticity may represent a previously unexplored target for the treatment of major depressive disorder.

STAR★METHODS

Detailed methods are provided in the online version of this paper and include the following:

- KEY RESOURCES TABLE
- RESOURCE AVAILABILITY
  - Lead contact
  - Materials availability
  - Data and code availability
- EXPERIMENTAL MODEL AND SUBJECT DETAILS
  - Animals
  - Primary neuronal culture
  - Cell lines
- METHOD DETAILS
  - Constructs for knockdown and gene expression
  - Lentiviral preparation
  - Primary neuron transfection
  - Spine analysis
  - Immunostaining and analysis
  - Whole-cell patch-clamp recording
  - Field recording
  - Drug injection
  - Forced swim test
  - Sucrose preference test after chronic restraint stress

(K) Distribution of individual GluA2 puncta size (vehicle, n = 4,932; ketamine, n = 4335; ketamine + anisomycin, n = 4,651 from 4 mice in each condition). The histogram was fitted by Gaussian distribution (vehicle, mean + 3 SD = 0.246 μm^2; ketamine, mean + 3 SD = 0.238 μm^2, ketamine + anisomycin, mean + 3 SD = 0.257 μm^2).

(L) Similar to surface GluA1, surface GluA2 was increased multiplicatively in CA1 stratum radiatum of mice treated with ketamine but anisomycin suppressed this scaling (linear regression, slope = 1.16 in ketamine and = 1.05 in ketamine + anisomycin, Mann-Whitney test, $p < 0.0001$ in ketamine compared to ketamine + anisomycin, n = 3,779).

See also Figure S7.

- ○ Novelty-suppressed feeding test
- ○ Immunohistochemistry and quantification for surface GluA1 and GluA2
- ○ Protein quantification
- ○ RNA extraction and RT-PCR
- ● QUANTIFICATION AND STATISTICAL ANALYSIS
  - ○ Statistical analysis

SUPPLEMENTAL INFORMATION

Supplemental information can be found online at https://doi.org/10.1016/j.celrep.2021.109918.

ACKNOWLEDGMENTS

We thank all of the members of the Monteggia and Kavalali laboratories for their support and discussions. We thank Drs. A. Ryazanov (University of Medical and Dentistry of New Jersey) for the eEF2K-KO mice, L. Chen (Stanford University) for the RARα$^{fl/fl}$ mice, and T. Südhof (Stanford University) for the L307 vector. This work was supported by National Institutes of Health grants MH070727 and MH081060 (to L.M.M.) and MH066198 (to E.T.K.), as well as an award from the Brain and Behavior Research Foundation (to K.S).

AUTHOR CONTRIBUTIONS

K.S. performed the molecular, biochemical, electrophysiological, and immunohistological experiments. E.N. and J.-W.K. performed the field recordings. K.S. and J.-W.K. performed the behavioral experiments. K.S., E.T.K., and L.M.M were responsible for the experimental design and wrote the paper. E.T.K. and L.M.M. supervised this project.

DECLARATION OF INTERESTS

The authors declare no competing interests.

Received: January 26, 2021
Revised: May 10, 2021
Accepted: October 11, 2021
Published: November 2, 2021

REFERENCES

Abdallah, C.G., Salas, R., Jackowski, A., Baldwin, P., Sato, J.R., and Mathew, S.J. (2015). Hippocampal volume and the rapid antidepressant effect of ketamine. J. Psychopharmacol. *29*, 591–595.

Adaikkan, C., Taha, E., Barrera, I., David, O., and Rosenblum, K. (2018). Calcium/Calmodulin-Dependent Protein Kinase II and Eukaryotic Elongation Factor 2 Kinase Pathways Mediate the Antidepressant Action of Ketamine. Biol. Psychiatry *84*, 65–75.

Adesnik, H., Li, G., During, M.J., Pleasure, S.J., and Nicoll, R.A. (2008). NMDA receptors inhibit synapse unsilencing during brain development. Proc. Natl. Acad. Sci. USA *105*, 5597–5602.

Aoto, J., Nam, C.I., Poon, M.M., Ting, P., and Chen, L. (2008). Synaptic signaling by all-trans retinoic acid in homeostatic synaptic plasticity. Neuron *60*, 308–320.

Autry, A.E., Adachi, M., Nosyreva, E., Na, E.S., Los, M.F., Cheng, P.F., Kavalali, E.T., and Monteggia, L.M. (2011). NMDA receptor blockade at rest triggers rapid behavioural antidepressant responses. Nature *475*, 91–95.

Berman, R.M., Cappiello, A., Anand, A., Oren, D.A., Heninger, G.R., Charney, D.S., and Krystal, J.H. (2000). Antidepressant effects of ketamine in depressed patients. Biol. Psychiatry *47*, 351–354.

Chen, N., and Napoli, J.L. (2008). All-trans-retinoic acid stimulates translation and induces spine formation in hippocampal neurons through a membrane-associated RARalpha. FASEB J. *22*, 236–245.

Chen, R.Z., Akbarian, S., Tudor, M., and Jaenisch, R. (2001). Deficiency of methyl-CpG binding protein-2 in CNS neurons results in a Rett-like phenotype in mice. Nat. Genet. *27*, 327–331.

Chu, H.P., Liao, Y., Novak, J.S., Hu, Z., Merkin, J.J., Shymkiv, Y., Braeckman, B.P., Dorovkov, M.V., Nguyen, A., Clifford, P.M., et al. (2014). Germline quality control: eEF2K stands guard to eliminate defective oocytes. Dev. Cell *28*, 561–572.

Cull-Candy, S., Kelly, L., and Farrant, M. (2006). Regulation of Ca2+-permeable AMPA receptors: synaptic plasticity and beyond. Curr. Opin. Neurobiol. *16*, 288–297.

Daly, E.J., Singh, J.B., Fedgchin, M., Cooper, K., Lim, P., Shelton, R.C., Thase, M.E., Winokur, A., Van Nueten, L., Manji, H., and Drevets, W.C. (2018). Efficacy and Safety of Intranasal Esketamine Adjunctive to Oral Antidepressant Therapy in Treatment-Resistant Depression: A Randomized Clinical Trial. JAMA Psychiatry *75*, 139–148.

Dull, T., Zufferey, R., Kelly, M., Mandel, R.J., Nguyen, M., Trono, D., and Naldini, L. (1998). A third-generation lentivirus vector with a conditional packaging system. J. Virol. *72*, 8463–8471.

Gideons, E.S., Kavalali, E.T., and Monteggia, L.M. (2014). Mechanisms underlying differential effectiveness of memantine and ketamine in rapid antidepressant responses. Proc. Natl. Acad. Sci. USA *111*, 8649–8654.

Gideons, E.S., Lin, P.Y., Mahgoub, M., Kavalali, E.T., and Monteggia, L.M. (2017). Chronic lithium treatment elicits its antimanic effects via BDNF-TrkB dependent synaptic downscaling. eLife *6*, e25480.

Hanse, E., Seth, H., and Riebe, I. (2013). AMPA-silent synapses in brain development and pathology. Nat. Rev. Neurosci. *14*, 839–850.

Höflich, A., Kraus, C., Pfeiffer, R.M., Seiger, R., Rujescu, D., Zarate, C.A., Jr., Kasper, S., Winkler, D., and Lanzenberger, R. (2021). Translating the immediate effects of S-Ketamine using hippocampal subfield analysis in healthy subjects-results of a randomized controlled trial. Transl. Psychiatry *11*, 200.

Huang, Y.C., and Cheng, Y.C. (2017). Isotretinoin treatment for acne and risk of depression: a systematic review and meta-analysis. J. Am. Acad. Dermatol. *76*, 1068–1076.e9.

Ionescu, D.F., Felicione, J.M., Gosai, A., Cusin, C., Shin, P., Shapero, B.G., and Deckersbach, T. (2018). Ketamine-Associated Brain Changes: A Review of the Neuroimaging Literature. Harv. Rev. Psychiatry *26*, 320–339.

Isaac, J.T., Ashby, M.C., and McBain, C.J. (2007). The role of the GluR2 subunit in AMPA receptor function and synaptic plasticity. Neuron *54*, 859–871.

Jacobs, D.G., Deutsch, N.L., and Brewer, M. (2001). Suicide, depression, and isotretinoin: is there a causal link? J. Am. Acad. Dermatol. *45*, S168–S175.

Jick, S.S., Kremers, H.M., and Vasilakis-Scaramozza, C. (2000). Isotretinoin use and risk of depression, psychotic symptoms, suicide, and attempted suicide. Arch. Dermatol. *136*, 1231–1236.

Kavalali, E.T., and Monteggia, L.M. (2020). Targeting Homeostatic Synaptic Plasticity for Treatment of Mood Disorders. Neuron *106*, 715–726.

Kerchner, G.A., and Nicoll, R.A. (2008). Silent synapses and the emergence of a postsynaptic mechanism for LTP. Nat. Rev. Neurosci. *9*, 813–825.

Lally, N., Nugent, A.C., Luckenbaugh, D.A., Niciu, M.J., Roiser, J.P., and Zarate, C.A., Jr. (2015). Neural correlates of change in major depressive disorder anhedonia following open-label ketamine. J. Psychopharmacol. *29*, 596–607.

Lane, M.A., and Bailey, S.J. (2005). Role of retinoid signalling in the adult brain. Prog. Neurobiol. *75*, 275–293.

Lee, H.K. (2012). Ca-permeable AMPA receptors in homeostatic synaptic plasticity. Front. Mol. Neurosci. *5*, 17.

Li, N., Lee, B., Liu, R.J., Banasr, M., Dwyer, J.M., Iwata, M., Li, X.Y., Aghajanian, G., and Duman, R.S. (2010). mTOR-dependent synapse formation underlies the rapid antidepressant effects of NMDA antagonists. Science *329*, 959–964.

Li, C., Chen, J., Wang, W., Ai, M., Zhang, Q., and Kuang, L. (2019). Use of isotretinoin and risk of depression in patients with acne: a systematic review and meta-analysis. BMJ Open *9*, e021549.

Liao, D., Zhang, X., O'Brien, R., Ehlers, M.D., and Huganir, R.L. (1999). Regulation of morphological postsynaptic silent synapses in developing hippocampal neurons. Nat. Neurosci. *2*, 37–43.

Liu, S.J., and Zukin, R.S. (2007). Ca2+-permeable AMPA receptors in synaptic plasticity and neuronal death. Trends Neurosci. *30*, 126–134.

Maeng, S., Zarate, C.A., Jr., Du, J., Schloesser, R.J., McCammon, J., Chen, G., and Manji, H.K. (2008). Cellular mechanisms underlying the antidepressant effects of ketamine: role of alpha-amino-3-hydroxy-5-methylisoxazole-4-propionic acid receptors. Biol. Psychiatry *63*, 349–352.

Maghsoodi, B., Poon, M.M., Nam, C.I., Aoto, J., Ting, P., and Chen, L. (2008). Retinoic acid regulates RARalpha-mediated control of translation in dendritic RNA granules during homeostatic synaptic plasticity. Proc. Natl. Acad. Sci. USA *105*, 16015–16020.

Magin, P., Pond, D., and Smith, W. (2005). Isotretinoin, depression and suicide: a review of the evidence. Br. J. Gen. Pract. *55*, 134–138.

Mahgoub, M., Adachi, M., Suzuki, K., Liu, X., Kavalali, E.T., Chahrour, M.H., and Monteggia, L.M. (2016). MeCP2 and histone deacetylases 1 and 2 in dorsal striatum collectively suppress repetitive behaviors. Nat. Neurosci. *19*, 1506–1512.

Nosyreva, E., Szabla, K., Autry, A.E., Ryazanov, A.G., Monteggia, L.M., and Kavalali, E.T. (2013). Acute suppression of spontaneous neurotransmission drives synaptic potentiation. J. Neurosci. *33*, 6990–7002.

Nosyreva, E., Autry, A.E., Kavalali, E.T., and Monteggia, L.M. (2014). Age dependence of the rapid antidepressant and synaptic effects of acute NMDA receptor blockade. Front. Mol. Neurosci. *7*, 94.

Poon, M.M., and Chen, L. (2008). Retinoic acid-gated sequence-specific translational control by RARalpha. Proc. Natl. Acad. Sci. USA *105*, 20303–20308.

Price, R.B., Nock, M.K., Charney, D.S., and Mathew, S.J. (2009). Effects of intravenous ketamine on explicit and implicit measures of suicidality in treatment-resistant depression. Biol. Psychiatry *66*, 522–526.

Rao, A., and Craig, A.M. (1997). Activity regulates the synaptic localization of the NMDA receptor in hippocampal neurons. Neuron *19*, 801–812.

Reese, A.L., and Kavalali, E.T. (2015). Spontaneous neurotransmission signals through store-driven Ca(2+) transients to maintain synaptic homeostasis. eLife *4*, e09262.

Reese, A.L., and Kavalali, E.T. (2016). Single synapse evaluation of the postsynaptic NMDA receptors targeted by evoked and spontaneous neurotransmission. eLife *5*, e21170.

Rodriguez, A., Ehlenberger, D.B., Dickstein, D.L., Hof, P.R., and Wearne, S.L. (2008). Automated three-dimensional detection and shape classification of dendritic spines from fluorescence microscopy images. PLoS ONE *3*, e1997.

Rogers, S., Rozman, P.A., Valero, M., Doyle, W.K., and Buzsaki, G. (2021). Mechanisms and plasticity of chemogenically induced interneuronal suppression of principal cells. Proc. Natl. Acad. Sci. USA *118*, e2014157118.

Ryazanov, A.G., Shestakova, E.A., and Natapov, P.G. (1988). Phosphorylation of elongation factor 2 by EF-2 kinase affects rate of translation. Nature *334*, 170–173.

Sarti, F., Schroeder, J., Aoto, J., and Chen, L. (2012). Conditional RARα knockout mice reveal acute requirement for retinoic acid and RARα in homeostatic plasticity. Front. Mol. Neurosci. *5*, 16.

Stewart, S.A., Dykxhoorn, D.M., Palliser, D., Mizuno, H., Yu, E.Y., An, D.S., Sabatini, D.M., Chen, I.S., Hahn, W.C., Sharp, P.A., et al. (2003). Lentivirus-delivered stable gene silencing by RNAi in primary cells. RNA *9*, 493–501.

Sutton, M.A., Ito, H.T., Cressy, P., Kempf, C., Woo, J.C., and Schuman, E.M. (2006). Miniature neurotransmission stabilizes synaptic function via tonic suppression of local dendritic protein synthesis. Cell *125*, 785–799.

Sutton, M.A., Taylor, A.M., Ito, H.T., Pham, A., and Schuman, E.M. (2007). Postsynaptic decoding of neural activity: eEF2 as a biochemical sensor coupling miniature synaptic transmission to local protein synthesis. Neuron *55*, 648–661.

Suzuki, K., and Monteggia, L.M. (2020). The role of eEF2 kinase in the rapid antidepressant actions of ketamine. Adv. Pharmacol. *89*, 79–99.

Suzuki, K., Nosyreva, E., Hunt, K.W., Kavalali, E.T., and Monteggia, L.M. (2017). Effects of a ketamine metabolite on synaptic NMDAR function. Nature *546*, E1–E3.

Turrigiano, G.G. (2008). The self-tuning neuron: synaptic scaling of excitatory synapses. Cell *135*, 422–435.

Turrigiano, G.G., Leslie, K.R., Desai, N.S., Rutherford, L.C., and Nelson, S.B. (1998). Activity-dependent scaling of quantal amplitude in neocortical neurons. Nature *391*, 892–896.

Wang, H.L., Zhang, Z., Hintze, M., and Chen, L. (2011). Decrease in calcium concentration triggers neuronal retinoic acid synthesis during homeostatic synaptic plasticity. J. Neurosci. *31*, 17764–17771.

Wołoszynowska-Fraser, M.U., Kouchmeshky, A., and McCaffery, P. (2020). Vitamin A and Retinoic Acid in Cognition and Cognitive Disease. Annu. Rev. Nutr. *40*, 247–272.

Zanos, P., Moaddel, R., Morris, P.J., Georgiou, P., Fischell, J., Elmer, G.I., Alkondon, M., Yuan, P., Pribut, H.J., Singh, N.S., et al. (2016). NMDAR inhibition-independent antidepressant actions of ketamine metabolites. Nature *533*, 481–486.

Zarate, C.A., Jr., Singh, J.B., Carlson, P.J., Brutsche, N.E., Ameli, R., Luckenbaugh, D.A., Charney, D.S., and Manji, H.K. (2006). A randomized trial of an N-methyl-D-aspartate antagonist in treatment-resistant major depression. Arch. Gen. Psychiatry *63*, 856–864.

STAR★METHODS

KEY RESOURCES TABLE

REAGENT or RESOURCE	SOURCE	IDENTIFIER
Antibodies		
Rabbit polyclonal anti-phospho-eEF2	Cell Signaling Technology	Cat# 2331; RRID:AB_10015204
Rabbit polyclonal anti-eEF2	Cell Signaling Technology	Cat# 2332; RRID:AB_10693546
Mouse monoclonal anti-MAP2	Abcam	Cat# ab33580; RRID:AB_776172
Mouse monoclonal anti-PSD-95	Thermo Fisher Scientific	Cat# MA1-046; RRID:AB_2092361
Mouse monoclonal anti-Tau1	Millipore	Cat# MAB3420; RRID:AB_11213630
Mouse monoclonal anti-synapsin-1	Millipore	Cat# MABN894
Rabbit polyclonal anti-GluA1	Millipore	Cat# ABN241; RRID:AB_2721164
Mouse monoclonal anti-GluA2	Millipore	Cat# MAB397; RRID:AB_2113875
Rabbit monoclonal anti-eEF2K	Abcam	Cat# ab45168; RRID:AB_732084
Rabbit monoclonal anti-BDNF	Abcam	Cat# ab108319; RRID:AB_10862052
Rabbit polyclonal anti-ERK1/2	Cell Signaling Technology	Cat# 4695; RRID:AB_390779
Rabbit monoclonal anti-phospho-ERK1/2	Cell Signaling Technology	Cat# 4370; RRID:AB_2315112
Rabbit monoclonal anti-GAPDH	Cell Signaling Technology	Cat# 2118; RRID:AB_561053
Chemicals, peptides, and recombinant proteins		
Cytosine Arabinoside (AraC)	Sigma-Aldrich	Cat# C1768
QX-314	Sigma-Aldrich	Cat# 552233
Tetrodotoxin (TTX)	Enzo Life Sciences	Cat# BML-NA120-0001
D(-)-2-Amino-5-phosphonopentanoic acid (AP-5)	Abcam	Cat# Ab120003
Picrotoxin (PTX)	Sigma-Aldrich	Cat# P1675
6-Cyano-7-nitroquinoxaline-2,3-dione disodium salthydrate (CNQX)	Sigma-Aldrich	Cat# C239
Ketamine	Hospira Zoetis	Cat# 00409-2053-10 Cat# 10004027
Retinoic acid	Sigma-Aldrich	Cat# R2625
A-484954	Sigma-Aldrich	Cat# SML0861
AM580	Cayman	Cat# 15261
NBQX	Abcam	Cat# ab120046
Anisomycin	Sigma-Aldrich	Cat# A9789
Critical commercial assays		
Plus Cy3 and TSA Fluorescein system	Perkin Elmer	Cat# NEL753001KT
Experimental models: Cell lines		
Human embryonic kidney-293 (HEK293) cells	ATCC	Catalog # CRL-1573; RRID: CVCL_0045
Experimental models: Organisms/strains		
Mouse: C57BL/6J	The Jackson Laboratory	Cat# JAX:000664; RRID:IMSR_JAX:000664
Mouse: eEF2K-KO	(Chu et al., 2014)	N/A
Mouse: RARα fl/fl	(Sarti et al., 2012)	N/A
Mouse: CaMKII-Cre93	(Chen et al., 2001)	N/A
Oligonucleotides		
Forward primer for *RARα* 5′-AGGGCTGTAAGGGCTTCTTC-3′	This paper	N/A
Reverse primer for *RARα* 5′-ACATGCCCACGTCGAAACAT-3′	This paper	N/A

(*Continued on next page*)

CellPress
OPEN ACCESS

Continued		
REAGENT or RESOURCE	SOURCE	IDENTIFIER
Forward primer for *Gapdh* 5′-AGGTCGGTGTGAACGGATTTG-3′	(Mahgoub et al., 2016)	N/A
Reverse primer for *Gapdh* 5′-TGTAGACCATGTAGTTGAGGTCA-3′	(Mahgoub et al., 2016)	N/A
Recombinant DNA		
L307-eEF2K-KD	This paper	N/A
L307-control	This paper	N/A
pFUW-GFP-Cre	(Gideons et al., 2017)	N/A
pFUW-GFP	(Gideons et al., 2017)	N/A
pRSV-REV	(Dull et al., 1998)	Addgene, Cat# 12253
pCMV-VSV-G	(Stewart et al., 2003)	Addgene, Cat# 8454
pMDLg/pRRE	(Dull et al., 1998)	Addgene, Cat# 12251
Software and algorithms		
pCLAMP	Molecular Devices	https://www.moleculardevices.com
Clampfit	Molecular Devices	https://www.moleculardevices.com
MiniAnalysis	Synaptosoft	http://www.synaptosoft.com/MiniAnalysis
NeuroStudio	(Rodriguez et al., 2008)	N/A
Fiji / ImageJ	NIH	https://imagej.nih.gov/ij/
Prism	GraphPad	https://www.graphpad.com/

RESOURCE AVAILABILITY

Lead contact

Further information and requests for resources and reagents should be directed to and will be fulfilled by the lead contact, Lisa M. Monteggia (lisa.monteggia@vanderbilt.edu).

Materials availability

This study did not generate new unique reagents.

Data and code availability

All data supporting the findings of this study are available from the lead author upon request. This paper does not report original code. Any additional information required to reanalyze the data reported in this paper is available from the lead contact upon request.

EXPERIMENTAL MODEL AND SUBJECT DETAILS

Animals

All animal procedures were performed in accordance with the guide for the care and use of laboratory animals and were approved by the Institutional Animal Care and Use Committee at UT Southwestern Medical Center and Vanderbilt University. Breeding colonies were maintained in animal facilities and mice were kept on a 12-hour light/dark cycle with access to food and water *ad libitum*. eEF2K-KO and littermate control wild-type mice were obtained from heterozygous eEF2K-KO crosses (Nosyreva et al., 2013). RAR$\alpha^{fl/fl}$ mice were generated as previously described and maintained as homozygous crosses (Sarti et al., 2012). The RARα-KO mice were generated by breeding transgenic mice expressing Cre recombinase under the control of the calcium/calmodulin-dependent kinase II promoter (CaMKII-Cre93 line) (Chen et al., 2001) to RAR$\alpha^{fl/fl}$ mice. Genotypes were confirmed by PCR analysis of genomic DNA from tails. C57BL/6 mice were habituated to the animal facility for 1 week before testing. 2 months old or older male mice were used for field recording and behavioral analysis.

Primary neuronal culture

Dissociated hippocampal cultures were prepared from whole hippocampi of mice (postnatal day 1-3) as previously described (Gideons et al., 2014; Suzuki et al., 2017). The hippocampi were trypsinized, dissociated mechanically and plated on Matrigel (Corning Inc, NY) coated coverslips. For suppression of glia cells, 2-3 μM cytosine arabinoside (AraC; Sigma) was added to culture media at days *in vitro* (DIV) 1 and concentration of AraC was reduced to half at DIV 4. Hippocampal neuronal cultures were maintained in 5% CO_2 at 37°C. For lentiviral gene expression and knockdown, neurons were infected with lentivirus at DIV 4. All experiments for

immunocytochemistry, morphology, electrophysiology and biochemistry were performed between DIV 14-21 unless otherwise described in the figure legend.

Cell lines

Human embryonic kidney-293 (HEK293-T) cells were used to produce lentiviruses. HEK293-T cell were maintained in 5% CO2 at 37°C in Dulbecco's Modified Eagle Medium supplemented with 10% FBS, penicillin, and streptavidin.

METHOD DETAILS

Constructs for knockdown and gene expression

For eEF2K knockdown experiments, short hairpin RNA (shRNA) was inserted into L307, a lentiviral transfer vector that includes the H1 promoter and IRES-GFP. The oligonucleotides encoding eEF2K shRNA sequence (GGAGACAACAGACTGCGATGA) correspond to mouse eEF2K (1932-1952). The control vector contained a non-targeting shRNA sequence (GCGCGATAGCGCTAATAATTT) commercially available from Sigma. The pFUW-GFP-Cre construct was used for Cre recombinase expression in RAR$\alpha^{fl/fl}$ hippocampal cultures to generate RARα-KO neurons and was compared to p-FUW-GFP expression as a control.

Lentiviral preparation

Lentiviruses were prepared by transfection of HEK293-T cells using Fugene 6 (Roche) with the plasmid of interest together with plasmids encoding viral packaging and coating proteins (pRSV-REV, pCMV-VSV-G, and pMDLg/pRRE) (Addgene). The culture medium was exchanged for neuronal growth media 1 day after transfection. The supernatant of neuronal growth media containing viruses were harvested 3 days after transfection. Using this approach, we consistently obtained infection frequencies approaching ~100%.

Primary neuron transfection

For sparse expression of constructs in cultured hippocampal neurons, the transfection was performed as previously described (Reese and Kavalali, 2016). Primary mouse hippocampal cultures were directly transfected at DIV 8-11 with either control or eEF2K-KD constructs using Lipofectamine 3000 (Thermo Fisher Scientific). After incubation of DNA-Lipofectamine complexes with neurons for 3 hours, culture media was removed and replaced with conditioned neuronal culture media. Transfected neurons could be visualized by their GFP expression from the transfected construct starting 3 days after transfection.

Spine analysis

Wild-type mouse hippocampal cultures were prepared and transfected with control or eEF2K-KD vectors. Cultured neurons were fixed using 4% PFA and 4% sucrose in phosphate buffer at DIV 18-19. After washing with PBS, cultures were mounted. Confocal z stack images of secondary or tertiary dendritic segments (0.5 μm interval, 5-11 images/stack) were acquired using a 63x objective with scan zoom of 4 on a Zeiss LSM 510. Images were obtained at 1.024 × 1.024 pixels resolution. All stacked images were projected into single planes by summation. Two dendrites from each neuron were analyzed for spine analysis. Quantitative analysis for dendritic spines was performed by NeuronStudio (CNIC).

Immunostaining and analysis

Staining for phosphorylated eEF2 was performed and modified as previously described (Autry et al., 2011). Cultured neurons were fixed by 4% paraformaldehyde (PFA) in phosphate buffer with 4% sucrose for 20 min at RT. After washing with PBS, endogenous peroxidase activity was quenched in 0.6% H_2O_2 in PBS and cells were permeabilized with 0.2% Triton X-100 in PBS. Cultured neurons were blocked in 1% BSA with 2% goat serum in PBS, and then incubated with primary antibodies in a blocking buffer for overnight at 4°C. Dilution of primary antibodies were as follows: 1:500 for rabbit anti-phospho-eEF2 (Cell signaling, 2331), 1:100 for rabbit anti-eEF2 (Cell signaling, 2332), 1:250 for mouse anti-MAP2 (Abcam, ab33580), 1:200 for mouse anti-PSD-95 (Thermo Scientific, MA1-046), 1:500 for mouse anti-Tau-1 (EMD Millipore, MAB3420) and 1:1000 for mouse anti-synapsin-I (EMD Millipore, MABN894). To detect phosphorylated eEF2 or total eEF2, anti-rabbit HRP antibody was applied at 1:1000 and the signal was amplified using the fluorescein or Cy3 tyramide amplification signal system (Perkin Elmer). Alexa 594 or 649-conjugated anti-mouse secondary antibodies were applied for anti-mouse primary antibodies. A post fixation was performed with 4% PFA in PBS.

Confocal fluorescent images were taken using a Zeiss LSM 510 or LSM 710 with a 63x objective at 1.024 × 1.024 pixels resolution (NA 1.4). Single confocal images were shown for identification and localization of phosphorylated eEF2 or total eEF2 (Figures S3A–S3D and S5A–S5D). To quantify relative eEF2 phosphorylation in the dendritic spine, GFP was expressed in the neurons as a volume maker. Confocal z stack images of dendrites were projected into a single image. PSD-95 immuno-reactivity (IR) and GFP IR in the dendritic spines were selected using the circular selection tool of ImageJ (National Institutes of Health), and then phosphorylated eEF2 IR was measured in the same region of interest (ROI). After background subtraction, phosphorylated eEF2 IR was normalized by GFP IR to consider as a relative eEF2 phosphorylation in spine. The values of phosphorylated eEF2-IR/GFP-IR and PSD-95-IR were further standardized using Excel as mean average is 0 and standard deviation is 1. All data were accumulated into scatterplot from several images of dendrites.

CellPress
OPEN ACCESS

Whole-cell patch-clamp recording

Whole-cell patch-clamp recordings were performed on hippocampal pyramidal neurons at DIV14-21 cultures. Data were acquired using a MultiClamp 700B amplifier and Clampex 10.0 software (Molecular Devices). Recordings were sampled at 100 μs and filtered at 2 kHz, with a gain of 5. Internal pipette solution contained 115 mM $CsMeSO_3$, 10 mM CsCl, 5 mM NaCl, 10 mM HEPES, 0.6 mM EGTA, 20 mM tetraethylammonium chloride, 4 mM Mg-ATP, 0.3 mM Na_3GTP, pH 7.3 and 10 mM QX-314 [N-(2,6-dimethylphenyl-carbamoylmethyl)-triethylammonium bromide], 300 mOsm. The external Tyrode's solution contained 150 mM NaCl, 4 mM KCl, 2 mM $CaCl_2$, 10 mM glucose, and 10 mM HEPES (pH 7.4), 310 mOsm. To isolate AMPA receptor miniature EPSC (AMPAR-mEPSC), the external $MgCl_2$ concentration was 1.25 mM and 1 μM tetrodotoxin (TTX, ENZO), 50 μM D(-)-2-Amino-5-phosphonopentanoic acid (AP-5, abcam) and 50 μM picrotoxin (PTX, Sigma) were added to bath solution. The holding potential was –70 mV. For whole-cell patch-clamp recordings in neurons transfected with construct, GFP was visualized with a GFP filter and X-cite 120 illumination (EXFO). To isolate AMPAR-mEPSCs and NMDAR-mEPSCs from a single neuron, AMPAR-mEPSCs were initially recorded at −70 mV as holding potential in the presence of 2 mM $MgCl_2$, 1 μM TTX, 50 μM PTX, 15 μM glycine and 1 μM strychnine in the external solution. After recording AMPAR-mEPSCs, we perfused the same external solution containing 10 μM 6-cyano-7-nitroquinoxaline-2,3-dione (CNQX, Sigma) to block AMPARs then, NMDA-mEPSC were recorded at +40 mV in the presence of CNQX. For evoked EPSC (eEPSC) recordings, field stimulation was applied through parallel bipolar electrodes using 35 mA of current. AMPAR-eEPSCs were isolated at −70 mV as holding potential in the external solution containing 2 mM $MgCl_2$ and 50 μM PTX, and AMPAR-eEPSC amplitude was determined by measuring the peak of response. After recording of AMPAR-eEPSC, NMDAR-eEPSC were obtained from recording at +40 mV, and measuring the amplitude of eEPSC 150 msec after EPSC onset. To induce synaptic scaling by blocking NMDAR under resting conditions, cultured hippocampal neurons were treated with 50 μM AP-5 or 50 μM ketamine in the presence of 1 μM TTX for 3 hours. For retinoic acid (RA)- or eEF2K inhibitor-mediated mediated synaptic scaling, cultured hippocampal neurons were treated with 1 μM RA or 30 μM A-484954 for 1 hour. No more than three recordings were obtained per coverslip. RA (Sigma), A-484954 (Sigma) and AM580 (Cayman) were dissolved in DMSO with the final concentration of DMSO in culture medium less than 0.1%.

For data analysis, all recordings were analyzed from 4-5 min recording using MiniAnalysis software (Synaptosoft) with the experimenter blind to genotype and drug condition. AMPAR-mEPSCs were identified with 5 pA detection threshold. NMDA-mEPSCs were analyzed at 10 pA detection threshold. Rank order plots for synaptic scaling analysis were built as previously described (Reese and Kavalali, 2015). For this analysis, 100 AMPAR-mEPSCs amplitudes were randomly selected from each recording since high frequency cells may skew amplitude comparisons by over representation. All selected amplitudes were pooled, sorted from smallest to largest and the rank order plot was built with an exclusion of 1% large amplitudes since such large amplitudes may be outliers and give an analysis error to scaling factor. The scaling factor was calculated by linear fitting of the data from drug treatment and vehicle conditions using Prism (GraphPad). eEPSCs were analyzed using pCLAMP10 software.

Field recording

Field recordings were performed using hippocampal slices as previously described (Nosyreva et al., 2013, 2014). Hippocampal slices (400 μm) were prepared from WT control and RARα-cKO mice. Mice were anesthetized with isoflurane and decapitated soon after the disappearance of corneal reflexes. The brain was removed, dissected, and then sliced in ice-cold dissection buffer containing 2.6 mM KCl, 1.25 mM NaH_2PO_4, 26 mM $NaHCO_3$, 0.5 mM $CaCl_2$, 5 mM $MgCl_2$, 212 mM sucrose, and 10 mM dextrose using a vibratome (VT 1000S, Leica). Area CA3 was surgically removed from each slice immediately after sectioning. The slices were transferred into a reservoir chamber filled with ACSF containing 124 mM NaCl, 5 mM KCl, 1.25 mM NaH_2PO_4, 26 mM $NaHCO_3$, 2 mM $CaCl_2$, 2 mM $MgCl_2$, and 10 mM dextrose. Slices were allowed to recover for 2–3 hours at 30°C. ACSF and dissection buffer were equilibrated with 95% O_2 and 5% CO_2. For recording, slices were transferred to a submerged recording chamber, maintained at 30°C, and perfused continuously with ACSF at a rate of 2–3 ml/min. Field potentials (FPs) were recorded with extracellular recording electrodes (1 MΩ) filled with ACSF and placed in stratum radiatum of area CA1. FPs were evoked by monophasic stimulation (duration, 200 μs) of Schaffer collateral/commissural afferents with a concentric bipolar tungsten stimulating electrode (Frederick Haer). Stable baseline responses were collected every 30 s using a stimulation intensity (10–30 μA), yielding 50%–60% of the maximal response. To induce synaptic potentiation by ketamine, 20 μM ketamine was applied for 30 min as previously described (Nosyreva et al., 2013, 2014). To confirm the effect of AM580 on FPs, 20 μM AM580 was applied for 2 hours. ACSF or 0.05% DMSO were used as a vehicle for ketamine or AM580. FPs were filtered at 2 kHz and digitized at 10 kHz on a personal computer using custom software (LabVIEW, National Instruments). Synaptic strength was measured as the initial slope (10%–40% of the rising phase) of the FP. The initial slopes of the FP were expressed as percentages of the preconditioned baseline average.

Drug injection

All drug injections were delivered intraperitoneally. The doses of drug were as follows: ketamine (Hospira or Zoetis) 5 mg/kg in saline, AM580 (Cayman) 20 mg/kg, in saline with 4% DMSO / 5% ethanol / 10% Cremophor EL. NBQX (Abcam) was used in 10 mg/kg. Anisomycin (Sigma) was used in 100 mg/kg. It was dissolved in HCl/saline and neutralized by NaOH.

Forced swim test

The forced swim test (FST) was performed as previously described (Autry et al., 2011; Gideons et al., 2014; Nosyreva et al., 2013). Mice were habituated to the testing facility for 1 hour. After injection of the drug, mice were placed in a glass 4 L beaker with 3 L of 23-24°C water for 6 min. Test sessions were video-recorded. The last 4 min of each trial were scored by an observer blinded to drug condition and genotype to determine the amount of time immobile.

Sucrose preference test after chronic restraint stress

Mice were singly housed, and two identical 50 mL tubes containing tap-water were given. Mice were subjected to restraint stress for 3-4 hours/day for 2 weeks. After 2 weeks of restraint stress, two bottles containing either tap water or 1% sucrose solution were provided, and sucrose preference was measured for 16 hours to examine the formation of anhedonia-like phenotype in the mice. Mice showing the anhedonia-like phenotype (< 80% sucrose preference) were treated with vehicle or AM580, and 2 hours later, sucrose preference was measured for an additional 16 hours. To remove confounding factor induced from biased spatial preference, the location of two bottles was changed at least 3 times.

Novelty-suppressed feeding test

The novelty-suppressed feeding (NSF) test was performed as previously described (Autry et al., 2011; Gideons et al., 2014; Nosyreva et al., 2013). Mice were food-deprived for 24 hours before the test and then habituated to the testing facility for 1 hour. A single food pellet was placed in the center of 42 × 42 cm open field. Each mouse was placed in a corner of open field and allowed to explore for up to 10 minutes. The trial ended when the mouse chewed a part of food pellet. To assess differences in appetite, the amount of food consumed in the home cage was measured in a 5 minutes period for each mouse.

Immunohistochemistry and quantification for surface GluA1 and GluA2

At 2 hours after injection of AM580 (20 mg/kg) or ketamine (5 mg/kg), mice were anesthetized with ketamine (100 mg/kg, intraperitoneally [i.p.])/xylazine (10 mg/kg, i.p.) and then transcardially perfused with ice-cold PBS followed by 4% PFA in phosphate buffer. The brains were removed, post-fixed and cryo-protected and frozen. The brains were cut into 25 μm-thick using cryostat. The section was blocked with 5% normal goat serum and 1% BSA without permeabilization. After blocking, surface AMPARs were labeled with primary antibody specific to an N-terminal extracellular epitope of GluA1 (1:400, Millipore ABN241) or GluA2 (1:100, Millipore, MAB397) and visualized with AlexaFluor 488-conjugated secondary antibody.

Confocal fluorescence z stack images (0.5 μm intervals, 10 images at scan zoom of 8) were taken at stratum radiatum of dorsal hippocampal CA1 in brain sections using a Zeiss LSM 510 with a 63x objective (NA 1.4, oil) at 1.504 × 1.504 pixels resolution. A total of 4 fields were obtained from each animal. For GluA1 or GluA2 puncta quantifications, imaging analysis was performed using ImageJ (Fiji). Z stack images were projected to single image by summation. Images were thresholded using Li's minimum cross entropy algorithm to exclude background. A fast fourier transform (FFT) band pass filter was applied to the image to enhance contrast. After this processing, adjacent puncta were segmented using interactive H-watershed program. Detection threshold was determined by Li's minimum cross entropy thresholding. Using binary image, regions of interest (ROIs) for individual punctum were determined by particle analysis. Area criteria was set to 0.05-1 μm^2 since theoretical lateral resolution in confocal image was 178 nm from calculation (0.51x λexc/NA = 0.51*488 nm/1.4). Puncta area size and total intensity were obtained from z stack image with background subtraction. For puncta size analysis, Gaussian distribution curve was generated from histogram. The value of Mean+3SD from Gaussian distribution was determined for considering the range of area for single postsynaptic structure. To compare total intensity of all selected puncta under the condition in rank ordered plot, the number of puncta was made the same using random function. These puncta total intensities were ranked from smallest to largest to build the rank order plot. 0%–90% fraction was shown in figure since higher intensities show supralinear or sublinear distribution. Representative images shown in figures were processed by median filter.

Protein quantification

To quantify protein levels, western blotting was carried out as previously described (Autry et al., 2011; Gideons et al., 2014; Suzuki et al., 2017). To measure protein level in cultured neurons, cultured hippocampal neurons were lysed in Laemmli sample buffer containing phosphatase and protease inhibitors (Roche). To quantify protein level in hippocampus, hippocampus was dissected and lysed using RIPA buffer [50 mM Tris, pH 7.4, 1% Igepal® CA-630, 0.1% SDS, 0.5% sodium deoxycholate, 4 mM EDTA, 150 mM NaCl, phosphatase and protease inhibitors (Roche)], and lysate was mixed with Laemmli sample buffer. Samples were boiled for 5 min at 95°C. Then samples were loaded onto SDS-PAGE gels and transferred to nitrocellulose membranes. Primary antibodies were incubated with membrane at following dilutions: 1:500 for anti-eEF2K (Abcam, ab45168), 1:5,000 for anti-phosphorylated eEF2 (Thr56) (Cell Signaling, 2331), 1:2,000 for anti-total eEF2 (Cell Signaling, 2332), 1:1,500 for anti-BDNF (Abcam, ab108319), 1:20,000 for anti-ERK1/2 (Cell Signaling, 4695), 1:20,000 for anti-phospho-ERK1/2 (Cell Signaling, 4370) and 1:50,000 for anti-GAPDH (Cell Signaling, 2118). Membranes were incubated with HRP anti-rabbit secondary antibody (Vector Laboratories, Inc.). Chemiluminescence was detected using Clarity Western ECL substrate (Bio-Rad). Band intensities were analyzed by ImageJ

RNA extraction and RT-PCR

To confirm whether RARα expression is reduced by Cre lentivirus infection in cultured floxed RARα hippocampal neurons, mRNA was isolated and RT-PCR was performed. Cultured hippocampal neurons were lysed and RNA was collected using the PureLink RNA mini kit (Thermo Fisher Scientific). cDNA was synthesized by using random primer and SuperScript® III Reverse Transcriptase (Thermo Fisher Scientific). Using cDNA as a template, transcripts for *RARα* and *Gapdh* were amplified using Power SYBR Green PCR master mix (Applied Biosystems) in a 7500 Real-Time PCR system (Applied Biosystems). Primers are for 5′-AGGGCTG TAAGGGCTTCTTC-3′ and 5′-ACATGCCCACGTCGAAACAT-3′ for *RARα* 5′-AGG TCG GTG TGA ACG GAT TTG-3′ and 5′-TGT AGA CCA TGT AGT TGA GGT CA-3′ for *Gapdh*.

QUANTIFICATION AND STATISTICAL ANALYSIS

Statistical analysis

Statistical analyses were performed using Prism (GraphPad Software). All data are reported as mean ± SEM. An unpaired two-tailed t test or Mann-Whitney test was used for comparison when comparing two groups. One-way or two-way ANOVAs with multiple comparisons were performed when comparing three or more groups. Sidak, Dunnett, Tukey and Fisher LSD tests were used post hoc comparisons following ANOVA. Log-rank test was used for comparison of survival distribution. The Grubbs test was used when appropriate to identify and remove significant outliers. Statistical significance was defined as $*p < 0.05$; $**p < 0.01$; $***p < 0.001$; $****p < 0.0001$.

Cell Reports, Volume *37*

Supplemental information

Convergence of distinct signaling pathways on synaptic scaling to trigger rapid antidepressant action

Kanzo Suzuki, Ji-Woon Kim, Elena Nosyreva, Ege T. Kavalali, and Lisa M. Monteggia

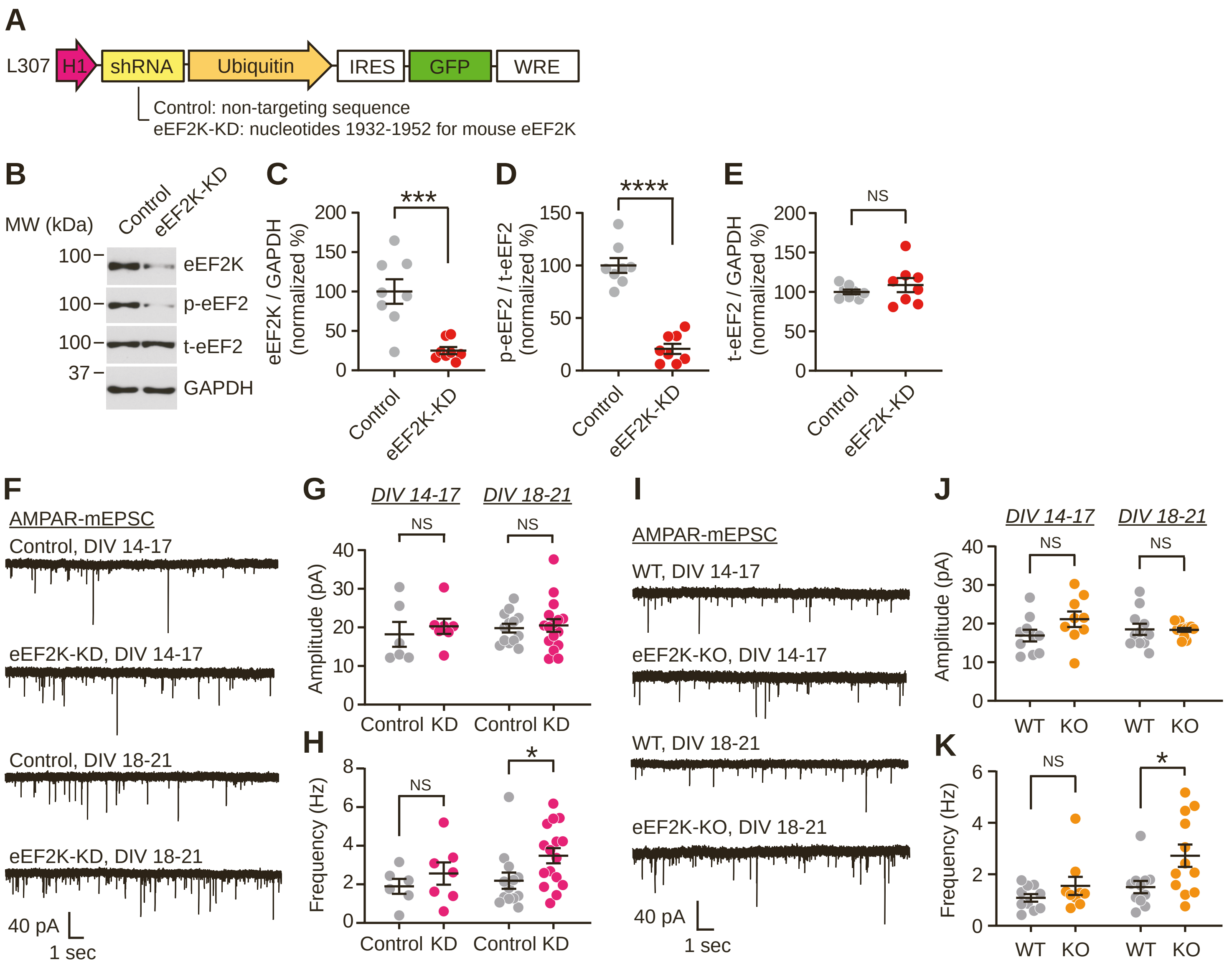

Fig. S1. Characterization of eEF2K knockdown and the effect on AMPAR-mEPSCs in cultured hippocampal neurons, related to Figure 1.

(A) Diagram of knockdown construct for eEF2K. shRNA sequence for corresponding to nucleotides 1932-1952 against mice eEF2K was subcloned into L307 vector. Non-targeting shRNA sequence was used as a control construct. (B) To confirm knockdown efficiency of eEF2K knockdown construct, control or eEF2K-KD lentivirus were infected in cultured hippocampal neurons at DIV4. Cell lysate was made at DIV16-17 and western blotting (WB) was performed using antibodies against eEF2K, phosphorylated eEF2 (p-eEF2) and total eEF2 (t-eEF2). (C) eEF2K expression was significantly decreased to 25.1 % ± 4.5% by eEF2K-KD. (Unpaired t test, P=0.0004, n=8 in control and eEF2K-KD). (D) Phospho-eEF2 level was strongly decreased to 20.7% ± 4.8% by eEF2K-KD since eEF2 is unique substrate for eEF2K (Unpaired t test, P<0.0001, n=8 in control and eEF2K-KD). (E), Total eEF2 level was not changed by eEF2K-KD (Unpaired t test, P=0.367, n=8 in control and eEF2K-KD). (F) Representative traces of AMPA-mEPSC from cultured hippocampal neurons infected with lentivirus control or eEF2K- KD. The ages of culture are indicated. (G) AMPAR-mEPSC amplitude was similar between control and eEF2K-KD neurons in younger (DIV 14-17) and older (DIV 18-21) mature cultured hippocampal neurons (Unpaired t test, P=0.5798, n=6 in control neurons and n=7 in eEF2K-KD neurons at DIV 14-17; P=0.7437, n=13 in control neurons and 16 in eEF2K-KD neurons at DIV 18-21). (H) A significant increase of frequency was not observed in eEF2K-KD young culture but older eEF2K-KD neurons augmented frequency (Unpaired t test, P=0.3763, n=6 in control neurons and n=7 in eEF2K-KD neurons at DIV 14-17; P=0.0329, n=13 in control neurons and n=16 in eEF2K-KD neurons at DIV 18-21). (I) Representative traces of AMPAR-mEPSC from WT and eEF2K-KO cultured hippocampal neurons. The ages of culture are indicated. (J) AMPAR-mEPSC amplitude was similar between WT and eEF2K-KO neurons at DIV 14-17 and DIV 18-21 (Unpaired t test, P=0.1085, n=10 in WT and n=9 in eEF2K-KO neurons at DIV 14-17; P=0.9297, n=11 in WT neurons and n=12 in eEF2K-KO neurons at DIV 18-21). (K) Older mature eEF2K-KO neurons, not young neurons, significantly increased AMPAR-mEPSC frequency (Unpaired t test, P=0.223, n=10 in WT and n=9 in eEF2K-KO neurons at DIV 14-17; P=0.0258, n=11 in WT neurons and n=12 in eEF2K-KO neurons at DIV 18-21). Data are represented as mean ±SEM.

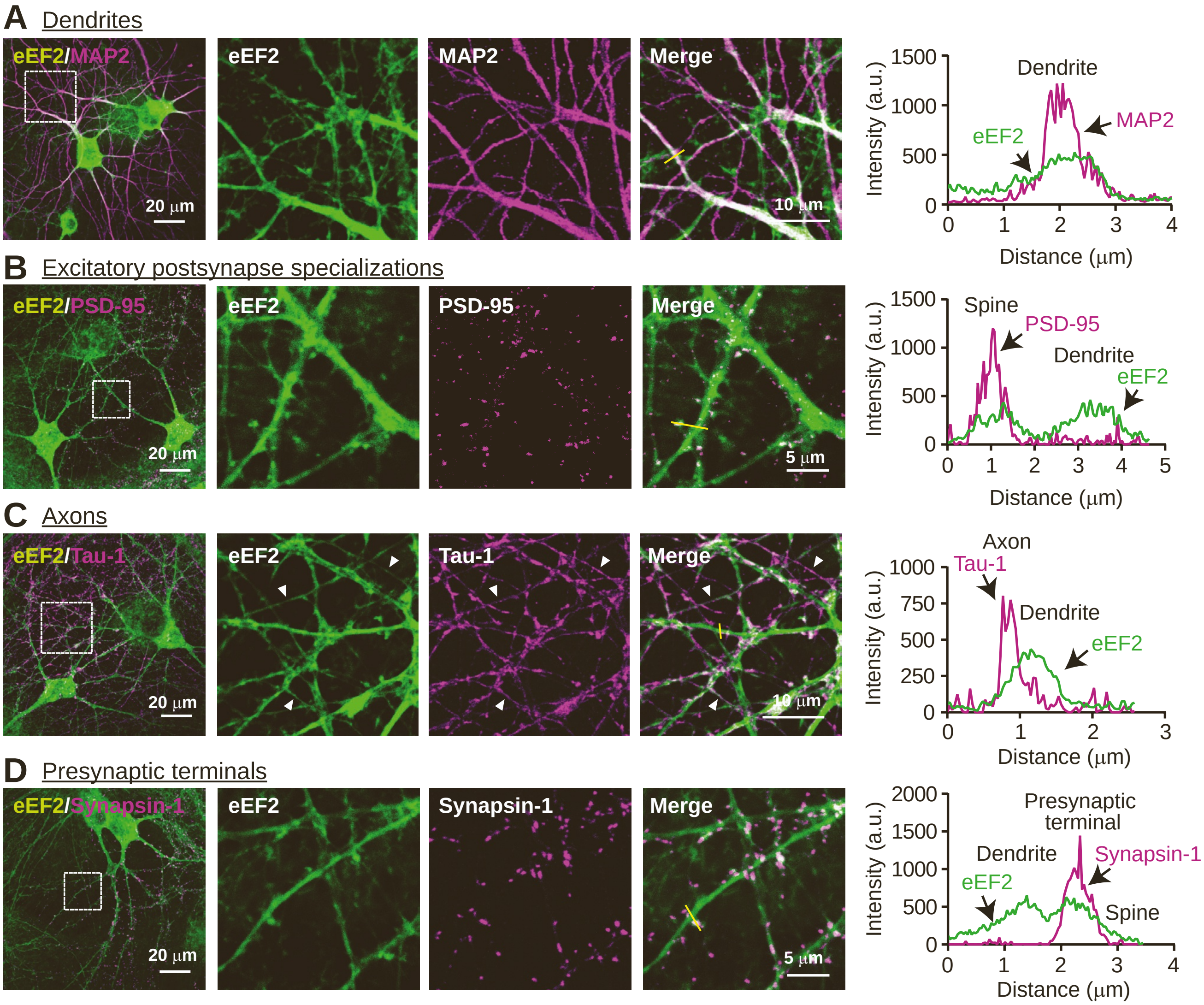

Fig. S2. Localization of eEF2 in cultured hippocampal neurons, related to Figure 1.
(A-D) To confirm the eEF2 distribution, immunostaining was performed using anti-total-eEF2. Left, Representative images of eEF2 with MAP2 (A), PSD-95 (B), tau-1 (C) or synapsin-1 (D) in mature cultured hippocampal neurons. Right, relative immunoreactivities (IR) on the yellow line in the merge figure (A-D). eEF2 was detected in dendrites and excitatory postsynapse specializations (A-B). eEF2 was observed in some axons (arrow head) but high eEF2 IR was detected in dendrites (C). Localization of eEF2 was not apparent in synapsin-I positive presynaptic terminal on dendrites (D). Scale bar, 20 μm, 10 μm or 5 μm.

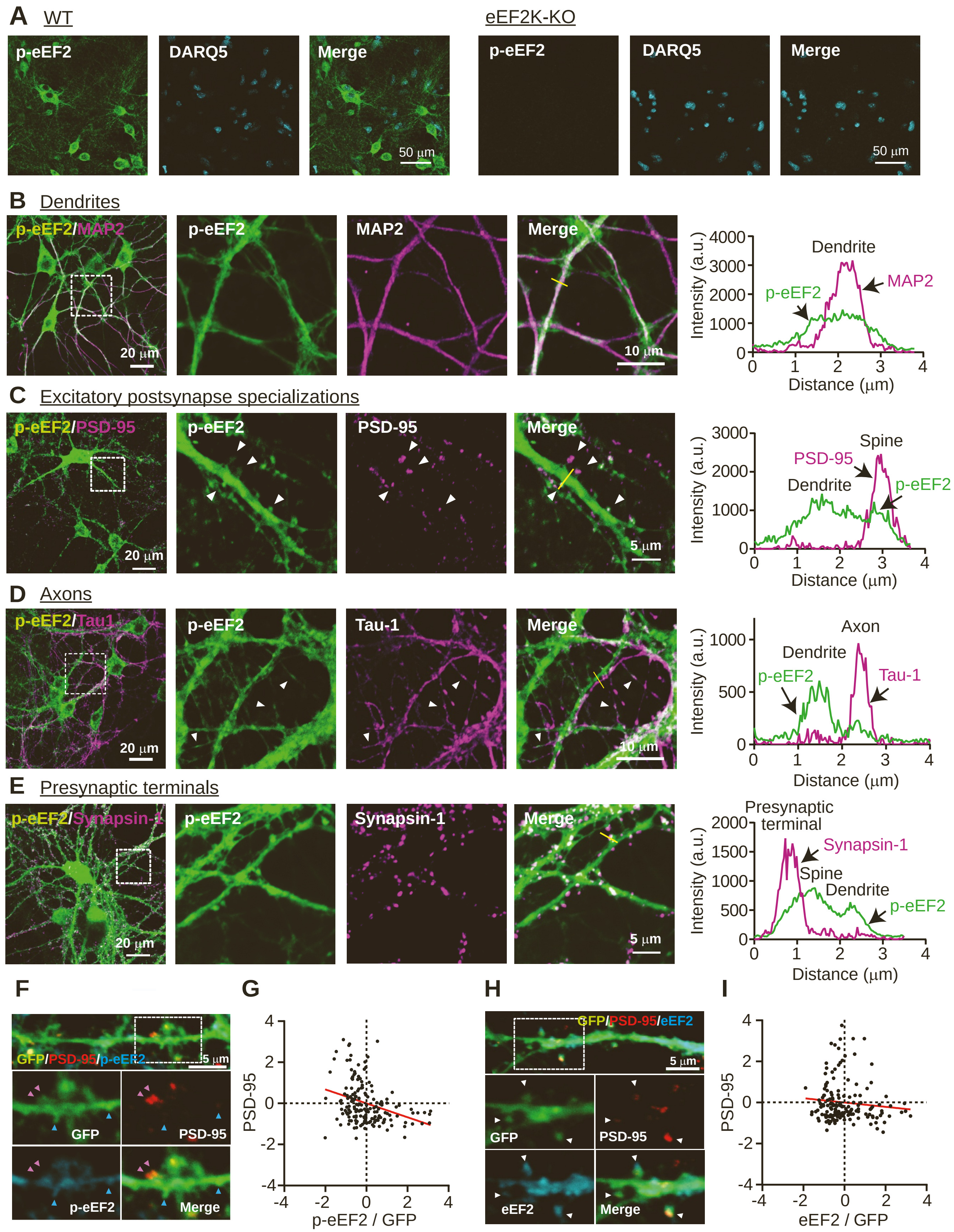

A
WT
p-eEF2
DARQ5
Merge
50 µm
eEF2K-KO
p-eEF2
DARQ5
Merge
50 µm
B
Dendrites
p-eEF2/MAP2
20 µm
p-eEF2
MAP2
Merge
10 µm
Dendrite
MAP2
p-eEF2
Intensity (a.u.)
Distance (µm)
C
Excitatory postsynapse specializations
p-eEF2/PSD-95
20 µm
p-eEF2
PSD-95
Merge
5 µm
Spine
PSD-95
Dendrite
p-eEF2
Intensity (a.u.)
Distance (µm)
D
Axons
p-eEF2/Tau1
20 µm
p-eEF2
Tau-1
Merge
10 µm
Axon
Dendrite
p-eEF2
Tau-1
Intensity (a.u.)
Distance (µm)
E
Presynaptic terminals
p-eEF2/Synapsin-1
20 µm
p-eEF2
Synapsin-1
Merge
5 µm
Presynaptic terminal
Synapsin-1
Spine
Dendrite
p-eEF2
Intensity (a.u.)
Distance (µm)
F
GFP/PSD-95/p-eEF2
5 µm
GFP
PSD-95
p-eEF2
Merge
G
PSD-95
p-eEF2 / GFP
H
GFP/PSD-95/eEF2
5 µm
GFP
PSD-95
eEF2
Merge
I
PSD-95
eEF2 / GFP

Fig. S3. eEF2 is phosphorylated in dendrites and excitatory postsynaptic spines, related to Figure 1.
(A) To confirm anti-phospho-eEF2 antibody specificity, immunostaining of cultured WT or eEF2K-KO hippocampal neurons was performed using antibody against phosphorylated eEF2 (p-eEF2). Immunoreactivity against p-eEF2 was abolished in eEF2K-KO hippocampal neurons. Nuclei were detected by DARQ5. Scale bar, 50 μm. (B-E) Left, Representative images of phosphorylated eEF2 (p-eEF2) with MAP2 (B), PSD-95 (C), tau-1 (D) or synapsin-1 (E) in mature cultured hippocampal neurons. Right, relative immunoreactivities (IR) on the yellow line in the merge figure (B-E). According to MAP2 or PSD-95 staining, eEF2 phosphorylation was observed in dendrites and excitatory postsynapse specializations (B-C). Unexpectedly, we observed variation of p-eEF2 IR and PSD-95 IR in synapses (arrow head in C). PSD-95 enrichment was detected in the spine showing lower eEF2 phosphorylation (C). Axons were identified using tau-1 staining in cultured hippocampal neurons. p-eEF2 IR was observed in some parts of axon (arrow head) (D). eEF2 phosphorylation was not apparent in synapsin-I positive presynaptic terminal on dendrites (E). Scale bar, 20 μm, 10 μm or 5 μm. (F) To closely analyze eEF2 phosphorylation level in the dendritic spines, we performed immunocytochemistry for phosphorylated (p-eEF2) with PSD-95 in the GFP-expressing hippocampal neurons. Several spines showed strong PSD-95 IR but they showed small p-eEF2 IR (pink arrow head). Conversely, small PSD-95 IR was observed in the spines showing high eEF2 phosphorylation (blue arrow head). Scale bar, 5 μm. (G) Relative eEF2 phosphorylation (p-eEF2/GFP) in the spines was determined by normalization of p-eEF2 IR with GFP IR as GFP is a volume marker. Then, individual p-eEF2/GFP and PSD-95 values were plotted in scatter plot as average is 0 and standard deviation is 1. Correlation analysis revealed small negative correlation between PSD-95 and relative p-eEF2 in the spines (Pearson correlation, r=-0.3378, P<0.0001 from 7 dendrites). (H) we performed same analysis using eEF2 staining to make sure whether this inverse correlation was occurred by eEF2 phosphorylation, not eEF2 expression. eEF2 was detected in PSD-95 positive spines (white arrow head). Scale bar, 5 μm. (I) PSD-95 intensities were not correlated with relative eEF2 intensities (Pearson correlation, r=-0.1049, P=0.1941 from 7 dendrites).

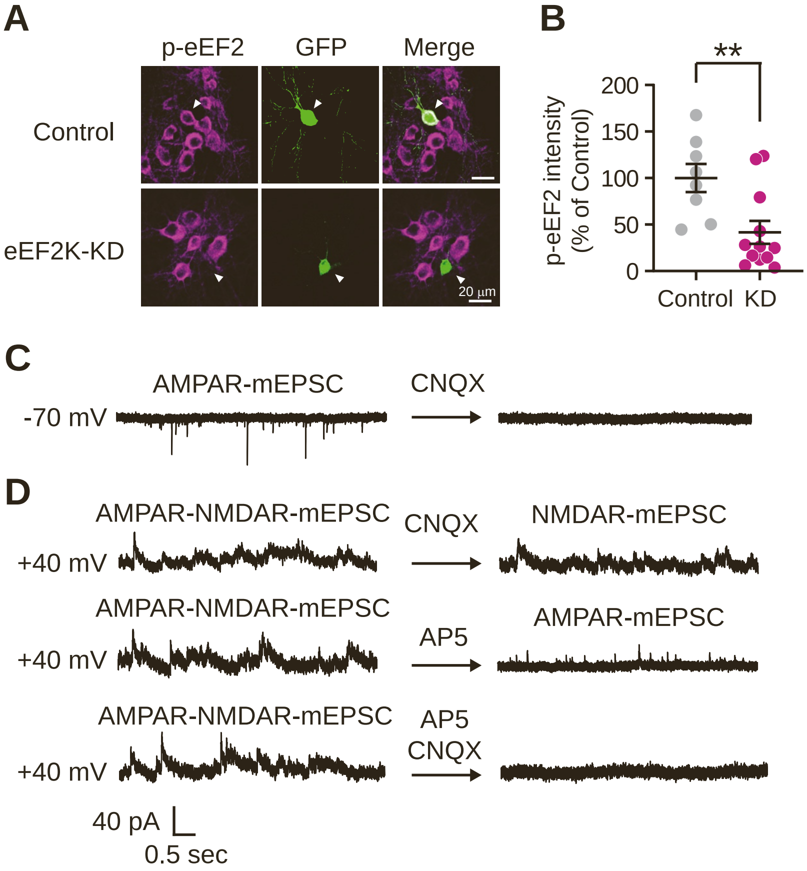

Fig. S4. Single cell knockdown for eEF2K and pharmacological isolation of AMPAR-mEPSCs and NMDAR-mEPSCs, related to Figure 1.

(A) Representative images of phosphorylated eEF2 (p-eEF2) in the cultured hippocampal neurons transfected with control or eEF2K-KD construct. Neurons were fixed at 3-4 day after transfection and immunocytochemistry for p-eEF2 was performed. p-eEF2 IR was reduced in the single cell eEF2K knockdown hippocampal neuron (arrow head). Scale bar, 20 µm. (B) p-eEF2 intensities in soma were measured in control or eEF2K-KD neurons. p-eEF2 signal was significantly reduced in the eEF2K-KD neurons (Unpaired t test, P=0.0077, n=8 in control and n=12 in eEF2K-KD). (C) AMPAR-mEPSC was isolated in the presence of Mg^{2+} at -70 mV since Mg^{2+} blocks NMDAR at -70 mV. Indeed, CNQX eliminated peaks of AMPAR-mEPSC. (D) AMPAR-NMDAR-mEPSC was recoding at +40 mV since Mg^{2+} is released from NMDAR at +40 mV. NMDA-mEPSC was isolated in the presence of CNQX. AMPAR-mEPSC was detected in the presence of AP-5 at +40 mV. Peaks were not detected in the presence of AP-5 and CNQX. Data are represented as mean ±SEM.

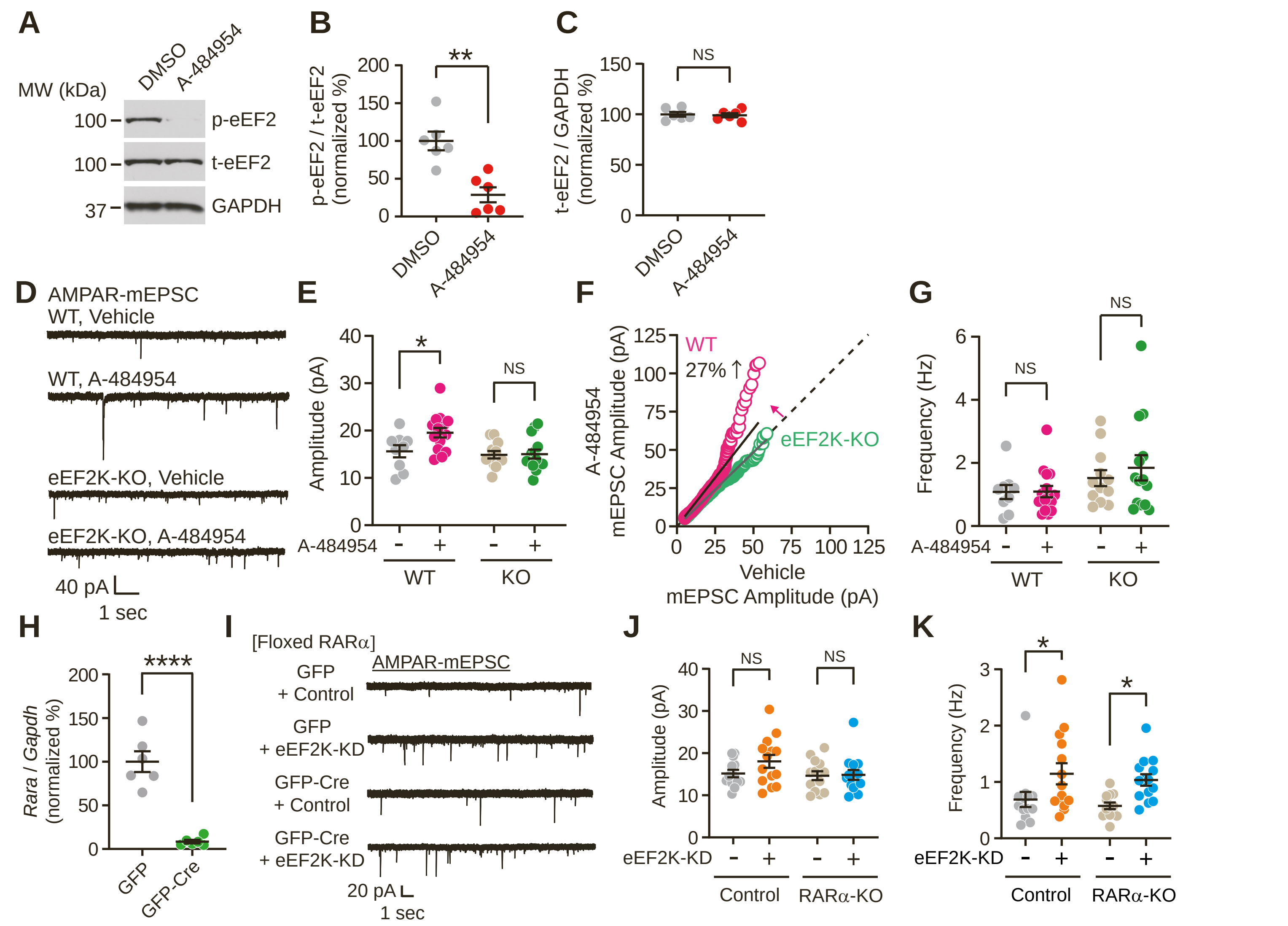

Fig. S5. Characterization of A484954-mediated synaptic scaling in eEF2K knockout neurons and the effect of loss of eEF2K on AMPAR-mEPSCs in RARα knockout neurons, related to Figure 2 and 3.
(A) To confirm whether A-484954 inhibits eEF2K activity in hippocampal neurons, we performed WB against phosphorylated eEF2 (p-eEF2) or total eEF2 (t-eEF2). After incubation of 30 μM A-484954 for 1 hour in wild type hippocampal culture, neuronal cells were lysed in lysis buffer and WB was performed using p-eEF2 and t-eEF2 antibodies. (B) Phosphorylated eEF2 was significantly reduced to 28.7 % ± 9.9% by A-484954 (Unpaired t test, P=0.0011, n=6 in control and eEF2K-KD). (C) Total EF2 level was not changed by A-484954 treatment (Unpaired t test, P=0.7719, n=6 in control and eEF2K-KD). (D) Representative traces of AMPAR-mEPSC from WT or eEF2K-KO cultured hippocampal neurons treated with vehicle or 30 μM A-484954 for 1 hour. (E) Acute treatment of A-484954 significantly increased AMPAR-mEPSC amplitude in WT neurons but eEF2K-KO neurons occluded it, suggesting that A-484954-mediated synaptic scaling acts through eEF2K dependent (Two-way ANOVA with Sidak's multiple comparisons, Vehicle vs A-484954 in WT P=0.0229, Vehicle vs A-484954 in eEF2K-KO P=0.9938, n=9-15 per group). (F) Rank order plot for AMPAR-mEPSC amplitude with or without A-484954 treatment in WT and eEF2K-KO neurons (Linear regression, slope= 1.27 in WT and =0.97 in eEF2K-KO). (G) Treatment of A-484954 did not change AMPAR-mEPSC frequency (Two-way ANOVA with Sidak's multiple comparisons, Vehicle vs A-484954 in WT P=0.9996, Vehicle vs A-484954 in eEF2K-KO P=0.6665, n=9-15 per group). (H) To confirm the RARα expression level in cultured floxed RARα hippocampal neurons with Cre lentivirus infection, mRNA was collected and RT-PCR was performed. RARα mRNA expression was significantly decreased to 8.445 ± 1.951 % by Cre lentivirus infection (Unpaired t test, P<0.0001, n=6 in GFP and GFP-Cre). (I) Effect of lentiviral eEF2K-KD on AMPAR-mEPSCs was confirmed in RARα-KO culture at DIV 18-21. (J) eEF2K-KD did not alter AMPAR-mEPSC amplitude in control and RARα-KO neurons (Two-way ANOVA with Sidak's multiple comparisons, control vs eEF2K-KD in control P=0.1824, control vs eEF2K-KD in RARα-KO P=0.9908, n=13-14 per group). (K) eEF2K-KD significantly increased AMPAR-mEPSC frequency even in RARα-KO neurons (Two-way ANOVA with Sidak's multiple comparisons, control vs eEF2K-KD in control P=0.0349, Control vs eEF2K-KD in RARα-KO P=0.0340, n=13-14 per group). Data are represented as mean ±SEM.

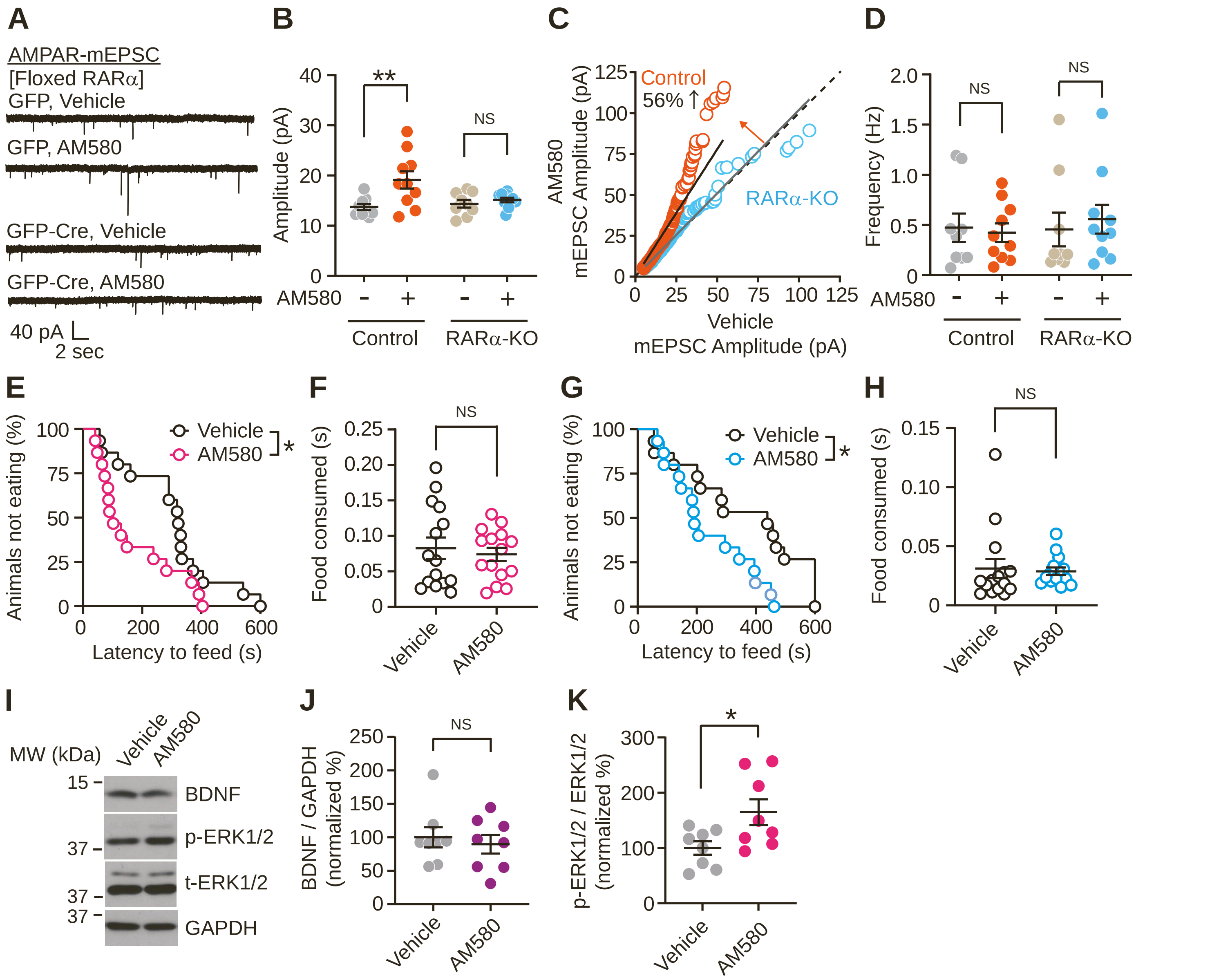

Fig. S6. The effect of RARα agonist AM580 on synaptic scaling, novelty-suppressed feeding and downstream signaling, related to Figure 4.

(A) Representative traces of AMPAR-mEPSC from control or RARα-KO cultured hippocampal neurons treated with vehicle or 10 μM AM580 for 1 hour. (B) Acute treatment of AM580 significantly increased AMPAR-mEPSC amplitude in control neurons but RARα-KO neurons occluded it, indicating AM580 specifically acts on RARα (Two-way ANOVA with Sidak's multiple comparisons, Vehicle vs AM580 in control *P*=0.0018, Vehicle vs AM580 in RARα-KO *P*=0.8507, *n*=9-10 per group). (C) Rank order plot for AMPAR-mEPSC amplitude with or without AM580 treatment in control and RARα-KO neurons (Linear regression, slope=1.56 in control and =1.02 in RARα-KO). (D) Treatment of A-484954 did not change AMPAR-mEPSC frequency (Two-way ANOVA with Sidak's multiple comparisons, Vehicle vs AM580 in control *P*=0.9592, Vehicle vs AM580 in RARα-KO *P*=0.8428, *n*=9-10 per group). (E) C57BL/6 mice were administered vehicle or AM580 (20 mg/kg) and assessed 2 hours later in novelty-suppressed feeding (NSF). AM580-treated C57BL/6 mice showed a significant decrease in latency to feed compared with vehicle (Log-rank test, *P*=0.0373, *n*=15 in vehicle and AM580). (F) appetite post-test following the NSF showed that the total amount of food consumed was similar between vehicle- and AM580-treated mice, ruling out a possible confound to the NSF test (Unpaired t test, *P*=0.6304, *n*=15 in vehicle and AM580). (G-H) 7 days after AM580 administration, AM580-treated mice displayed antidepressant-like response in the NSF (Log-rank test, *P*=0.0140, *n*=15 in vehicle and AM580), with no significant effect on food consumption test (Unpaired t test, *P*=0.7956, *n*=15 in vehicle and AM580). (I) To confirm whether AM580 increases BDNF or ERK phosphorylation in hippocampus, we quantified BDNF and ERK phosphorylation 2 hours after administration of vehicle or AM580 (20 mg/kg) in mice. Hippocampus was dissected and lysed in lysis buffer. WB was performed using antibodies [anti-BDNF, anti-phospho-EFK1/2 (p-ERK1/2), anti-total-ERK1/2 (t-ERK1/2) or anti-GAPDH]. (J) BDNF level was not altered by AM580 treatment (Unpaired t test, *P*=0.6242, *n*=8 in vehicle and AM580). (K) phosphorylation of ERK was significantly increased by AM580 treatment (Unpaired t test, *P*=0.0269, *n*=8 in vehicle and AM580). Data are represented as mean ±SEM.

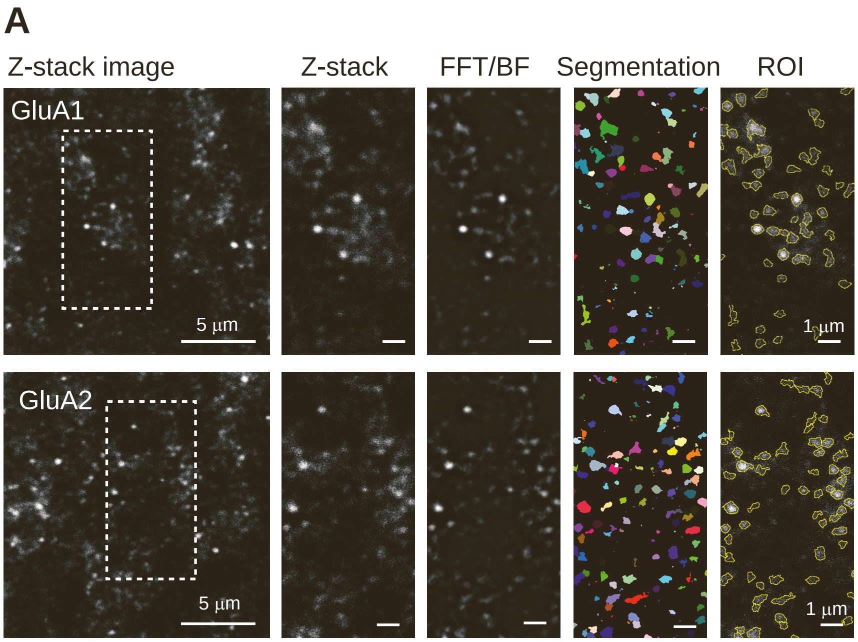

Fig. S7. Illustration of optical analysis for surface GluA1 and GluA2 puncta, related to Figure 5.
(A) To enhance particle contrast, surface GluA1 and GluA2 images of stratum radiatum of hippocampus CA1 region (as shown in Figure 5A and 5B) were processed using fast Fourier transform (FFT) bandpass filter (BF). To segment adjacent puncta, interactive H-watershed was applied on FFT/BF image. Regions of interest (ROIs) were determined using particle analysis. Area criteria was set to 0.05-1.00 μm^2. Area size and intensity in individual puncta were obtained from Z-stack image. Scale bar, 5 µm or 1 µm.
